# Disrupting the LINC complex by AAV mediated gene transduction prevents progression of Lamin induced cardiomyopathy

**DOI:** 10.1038/s41467-021-24849-4

**Published:** 2021-08-05

**Authors:** Ruth Jinfen Chai, Hendrikje Werner, Peter Yiqing Li, Yin Loon Lee, Khaing Thet Nyein, Irina Solovei, Tuan Danh Anh Luu, Bhavya Sharma, Raju Navasankari, Martina Maric, Lois Yu En Sim, Ying Jie Loh, Edita Aliwarga, Jason Wen Long Cheong, Alexandre Chojnowski, Matias Ilmari Autio, Yu Haiyang, Kenneth Kian Boon Tan, Choong Tat Keng, Shi Ling Ng, Wei Leong Chew, Michael Ferenczi, Brian Burke, Roger Sik Yin Foo, Colin L. Stewart

**Affiliations:** 1 Skin Research Institute (SRIS), Agency for Science, Technology and Research (A*STAR), Singapore, Singapore; 2 Cardiovascular Research Institute (CVRI), Yong Loo Lin School of Medicine, National University of Singapore, Singapore, Singapore; 3 Department of Biology II, Ludwig Maximilians University Munich, Munich, Germany; 4 Genome Institute of Singapore (GIS), Agency for Science, Technology and Research (A*STAR), Singapore, Singapore; 5 Muscle and Cardiac Biophysics Laboratory, Lee Kong Chien School of Medicine, Nanyang Technological University, Singapore, Singapore; 6 Deptartment of Physiology, School of Medicine, NUS, Singapore, Singapore

**Keywords:** Cytoskeletal proteins, Gene therapy, Cardiology

## Abstract

Mutations in the LaminA gene are a common cause of monogenic dilated cardiomyopathy. Here we show that mice with a cardiomyocyte-specific *Lmna* deletion develop cardiac failure and die within 3–4 weeks after inducing the mutation. When the same *Lmna* mutations are induced in mice genetically deficient in the LINC complex protein SUN1, life is extended to more than one year. Disruption of SUN1’s function is also accomplished by transducing and expressing a dominant-negative SUN1 miniprotein in *Lmna* deficient cardiomyocytes, using the cardiotrophic Adeno Associated Viral Vector 9. The SUN1 miniprotein disrupts binding between the endogenous LINC complex SUN and KASH domains, displacing the cardiomyocyte KASH complexes from the nuclear periphery, resulting in at least a fivefold extension in lifespan. Cardiomyocyte-specific expression of the SUN1 miniprotein prevents cardiomyopathy progression, potentially avoiding the necessity of developing a specific therapeutic tailored to treating each different *LMNA* cardiomyopathy-inducing mutation of which there are more than 450.

## Abstract (web summary)

Mutations in the LaminA gene are the second most common inherited cause of Dilated Cardiomyopathy, a major form of heart failure. Here the authors show that disruption of the nuclear protein SUN1 in cardiomyocytes, by AAV mediated transduction of a SUN1 inhibitor, significantly suppress cardiomyopathy progression, providing a potential therapeutic route to treat this disease.

## Introduction

Dilated Cardiomyopathy (DCM) is the most common disease affecting heart muscle, accounting for ~60% of all cardiomyopathies. It is characterized by reduced systolic (contractile) function due to enlargement and thinning of the left ventricular wall. In some cases, both ventricles are affected. DCM often results in sudden heart failure and cardiac death, resulting in high hospital admission rates, the need for heart transplantation, and consequently a high-cost burden^1,2^. The causes of DCM are varied but include various extrinsic factors (viral, autoimmune infiltration, lifestyle). However, 30–40% of all cases have a monogenic basis, with mutations in some 40 genes being linked to DCM. The most frequently mutated gene in DCM is *TTN*, which encodes the giant sarcomeric protein titin, with truncating variants in *TTN* resulting in almost 15–25% of all congenital forms of DCM^3,4^. The second most frequently mutated gene is Lamin A (*LMNA*) and accounts for as many as 6–8% of congenital DCM patients^4^.

*LMNA*-induced DCM is characterized by cardiac conduction defects, manifested by electrophysiological abnormalities, including atrioventricular block, ventricular arrhythmias, and fibrillation. The risk of sudden cardiac death is more significant in patients with *LMNA*-cardiomyopathy than patients with other forms of DCM^5^. Some 450 different dominant missense mutations have been identified in the *LMNA* gene that are linked to DCM. This diversity of mutations complicates genetic approaches to treating *LMNA*-induced DCM. To a limited extent, *LMNA*-linked DCM can be treated by fitting a pacemaker. Ultimately, effective treatment is accomplished by heart transplantation^6,7^.

Mice carrying *Lmna* mutations die within a few weeks after birth^8–11^. The cause of their early death is uncertain due to multiple tissues being affected. However, cardiac myopathy is considered to be a significant factor, as many *Lmna* mutant mice develop DCM with conduction abnormalities with focal myocyte degeneration^10,12^. Mice, therefore, provide a valuable model to determine the underlying molecular pathology of DCM.

The lamins are nuclear intermediate filament proteins and are the principal constituents of the nuclear lamina, the proteinaceous matrix underlying the inner nuclear membrane (INM). The lamina is composed of the A-type lamins, consisting of 2 predominant forms, lamins A and lamin C, derived by alternate splicing of *LMNA*. In addition, two B-type lamins (*LMNB 1* and *2*) are encoded by separate genes, *LMNB1* and *LMNB2*^13^. The lamina provides structural and mechanical integrity to the nucleus, maintains nuclear shape, and contributes to nuclear positioning within the cell. In addition, the lamina is a critical determinant of chromatin organization^8,14^, through the provision of binding sites at the nuclear periphery for higher-order chromatin domains. The lamins interact with numerous INM proteins, including Emerin, the Lamina-Associated Polypeptides (LAPs), and the SUN domain proteins^15^, many of which are either mutated or present as a variant linked to heart disease^16,17^. Together these proteins comprise an integrated protein network, centered on the lamina, where loss or mutation of the lamins can result in either the mislocalization or a change in their expression levels (emerin, SUN1, LBR, LAP1c, and Lap2 isoforms)^8,14,18–20^. Among the proteins, whose expression is altered by the loss or mutation of *Lmna* are SUN1 and LAP2α, whose levels increase. Genetic reduction of the levels of either protein, particularly SUN1, significantly ameliorates much of the pathology observed in mice with *Lmna* mutations, so increasing longevity^18,19^. Whether this is a consequence of SUN1 toxicity due to elevated expression levels versus elimination of a SUN1 function that exacerbates the effects of *Lmna* deficiency is uncertain.

The SUN (Sad1p, UNC-84) proteins share a conserved C-terminal SUN domain and localize to the INM^21^. In mammals, SUN1 and SUN2 are the two principal SUN proteins widely expressed in virtually all tissues. In the perinuclear space, between the INM and outer nuclear membrane (ONM), the C-termini of either SUN1 or 2 binds to the C-termini (KASH domains) of the different Nesprins/SYNE/KASH proteins that traverse the ONM. Together, these two families of proteins comprise the LINC complexes, which physically couple interphase nuclei to the cytoskeleton^22,23^. The N-termini of the SUN domain proteins protrude into the nucleoplasm. With SUN1, this region interacts with pre-laminA and nuclear pore complexes, as well as other nucleoplasmic/NE proteins such as DROSHA^24^. With the Nesprins/KASH-domain proteins, their large N-terminal domains extend into the cytoplasm adjacent to the ONM. Depending on the particular Nesprin/KASH protein, the Nesprins interact directly or indirectly with the three cytoskeletal protein networks (microtubules, actin microfilaments, and intermediate filaments)^25^. Consequently, the LINC complex establishes a direct physical connection between the cytoplasmic cytoskeletal networks (and their connections, e.g., cell adhesion complexes at the cell membrane) and the interphase nuclear interior or nucleoplasm. The LINC complex mediates force transmission between the nucleus and cytoskeleton, resulting in changes in gene expression/chromatin organization in response to mechanical/physical stimuli^26–28^. Although the loss of either SUN1 or SUN2 alone has no overt effect on postnatal growth and longevity, SUN1 null mice are both infertile and deaf. However, simultaneous loss of *SUN1 and SUN2* results in perinatal lethality, indicating some degree of redundancy^29^, at least during embryogenesis.

Here we show that in mice that develop Lmna-induced DCM, disruption of the LINC complex, by either genetic ablation or by delivery of a Dominantly Negative acting Sun1 miniprotein (DNSUN1) that destabilizes the LINC complex, significantly ameliorates DCM progression, and leads to at least a fivefold increase in longevity. We demonstrate that disruption of the LINC complex in individuals carrying *LMNA* mutations, AAV mediated delivery of a DN-SUN1 to CMs may be of therapeutic benefit to patients with *LMNA* associated DCM.

## Results

### Cardiomyocyte-specific loss of *Lmna* results in heart failure

**Fig. 1:**
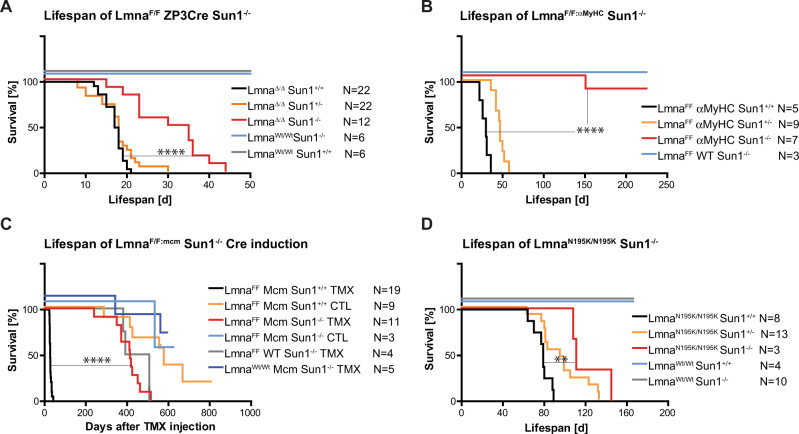
Loss of Sun1 extends the longevity of LaminA (*Lmna)* mutant mice. **A** The median postnatal lifespan of the *Lmna*^*F/F*^/*ZP3Cre* mice in which a conditional (floxed) *Lamin A* (*Lmna*^*F/F*^) is deleted in all tissues is 17.5 days. On a *Sun1*^*–/–*^ background, median longevity is increased to 32.5 days. **B** When *Lmna*^*F/F*^ is deleted specifically and constitutively in hearts by crossing the mice with the *αMyHC-Cre* line, the *Lmna*^*F/FːαMyHC*^*-Cre* mice have a median lifespan of 26.5 days. On a *Sun1*^*–/–*^ background, these mice live for longer than 6 months. **C** 3–5-month-old *Lmna*^*F/F*^ mice were crossed with the tamoxifen (Tmx) inducible cardiomyocyte-specific Cre Tg(*Myh6-Cre/Esr1*) (abbreviated to *mcm*), after a single injection of Tmx the mice die within 3–4 weeks. On a *Sun1*^*–/–*^ background, these mice (*Lmna*^*F/F:mcm*^) live for more than 1 year. **D** *Lmna*^*N195K/N195K*^ animals live for 78 days compared to *Lmna*^*N195K/N195K*^*Sun1*^*–/–*^ which had a median lifespan of 118 days. **A–D** (****P < 0.0001; **P = 0.0073 Log-rank (Mantel–Cox) test).

**Fig. 2:**
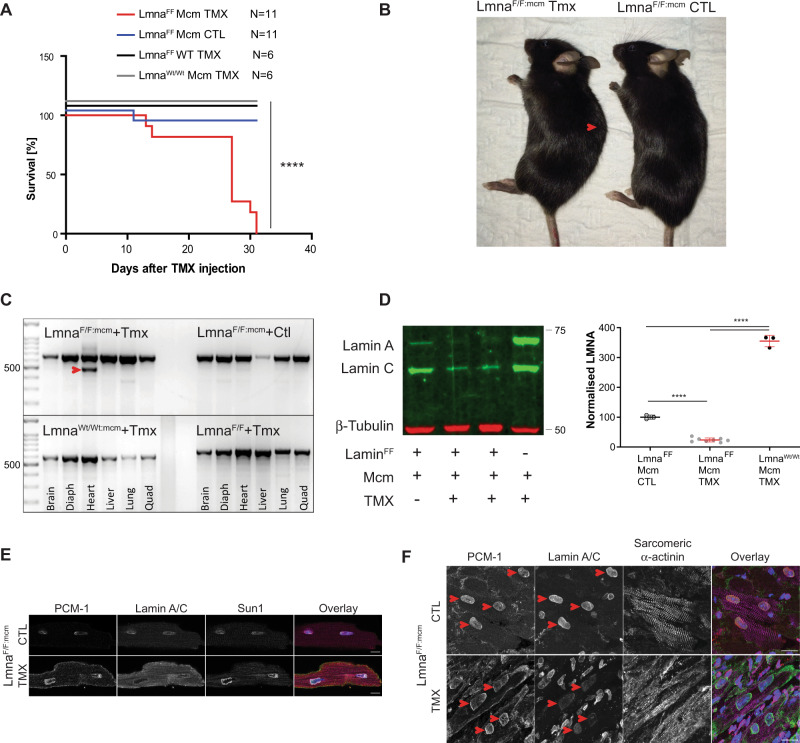
Lifespan and cardiac phenotype of the *Lmna*^*F/F:mcm*^ mice. **A** The median lifespan of the *Lmna*^*F/F:mcm*^ mice was 27 days after a single Tmx injection (****P < 0.0001; Log-rank (Mantel–Cox) test). **B** *Lmna*^*F/F:mcm*^ mice develop kyphosis (red arrow) by 21 days after injection. **C** PCR detected the floxed (deleted) *Lmna* gene (red arrowhead) only in whole heart tissue after Tmx induction and neither in other tissues or when Tmx was not injected. Assays were performed once with the number of animals as indicated in the graph. DNA marker in bp (**D**) LaminA/C levels were quantified by Western analysis of whole heart lysates 21 days after Tmx induction. A significant reduction in A-type Lamin protein was detected. Lamin C levels were not reduced as much in the *Lmna*^*F/F:mcm*^ hearts compared to *Lmna*^*F/F:mcm*^ controls (****P < 0.0001; unpaired two-tailed *T*-test, data presented as mean ± SD). Quantitative analysis was performed at 21 days post-Tmx induction (right panel). The presence of the *LoxP* sites in the WT-*Lmna* gene (*Lmna*^*F/F*^) results in a reduction in *Lmna* transcript levels compared to *Lmna*^*Wt/Wt*^ levels. However, this had no overt effect on longevity or postnatal growth/viability. Markers in kDA (**E**, **F**) LaminA/C protein, detected by immunofluorescence, was reduced/absent in CM nuclei in both (**E**) isolated CMs (Scale bar 10 μm) and (**F**) heart sections (red arrows) with CM nuclei detected by PCM-1 staining, 21 days after Tmx induction. Nuclei are in blue. The images shown are representative of three independently performed experiments. Scale bar 10 μm. Source data are provided as a Source Data file.

To further define the consequences of *Lmna* loss to postnatal pathology in mice, we specifically ablated the *Lmna* gene in specific tissues by using a floxed conditional *Lmna*^*F/F*^ line of mice that, when recombined by *Cre* activation, results in the complete loss of LaminA/C protein as previously described^11,14^. If the *Lmna*^*F/F*^ is constitutively deleted in all tissues by crossing the *Lmna*^*F/F*^ mice with *Zp3-Cre* mice^30^, the mean postnatal lifespan is 17.5 days (Fig. 1A). When the same deletion is induced in the absence of *Sun1*, *Lmna*^*∆/∆*^*:Sun1*^*−/−*^ mice lived to a mean of 32.5 days, almost a doubling in longevity (Fig. 1A). Performing the same *Lmna* deletion on a *Sun2* null background did not extend the longevity of *Lmna*^*∆/∆*^ mice, revealing the longevity extension is specific to the loss of *Sun1* even though SUN2 is expressed in multiple tissues, including cardiomyocytes (Supplementary Fig. 1). As the A-type lamins are widely expressed in almost all adult tissues, we then determined whether *Lmna* deletion, specifically in cardiomyocytes (CMs), contributes to the early postnatal death of *Lmna*^*∆/∆*^ mice and what consequences the same tissue-specific loss of *Lmna* would have on a *Sun1* null background. We crossed the *Lmna*^*F/F*^ to α*Myh6-Cre*^31^ mice, in which constitutive *Cre* expression commences during embryogenesis but is restricted to CMs. These mice survived slightly longer than the *Lmna*^*∆/∆*^ for an average of 26.5 days postnatally (Fig. 1B). The same CM-specific deletion performed on a *Sun1*^*−/−*^ background resulted in a significant increase in longevity to at least 6 months and beyond after birth (Fig. 1B), revealing that loss of SUN1 significantly extends longevity in mice with *Lmna*-induced heart failure. To further define the loss of *Lmna* and its effect in postnatal/adult CMs, we derived mice homozygous for the *Lmna*^*F/F*^ allele carrying the inducible cardiomyocyte-specific Cre-Tg (*Myh6-Cre/Esr1*) (abbreviated to *mcm*) in which *Cre* is induced in CMs by a single injection of tamoxifen (Tmx)^32^. The average lifespan of 1–5 month-old *Lmna*^*F/F:mcm*^ mice, following *Cre* induction, was 27 days (Figs. 1C, 2A). *Lmna*^*F/F*^ or WT controls were unaffected by Tmx injection and showed typical longevity. However, on a *Sun1* null background, longevity was significantly extended to more than 400 days following *Cre* induction (see below).

As most cases of *LMNA*-induced DCM result from missense mutations, we determined what effect loss of SUN1 had on the longevity and cardiac function of a previously described *Lmna* mutant mouse line, carrying the *N195K* missense mutation, in which early death is a consequence of heart failure^12^. This mutation has been identified in at least two unrelated patients diagnosed with DCM^33,34^. Here too, we found that the absence of SUN1 significantly increased the lifespan of this mutant line and improved cardiac function (Fig. 1D). We extended these findings by deriving mice heterozygous for the *N195K* mutation, where the WT-*Lmna* allele is floxed, i.e., *Lmna*^*N195K/F *^ *X* *Sun1*^*+/+*^. Activating Cre in these mice by Tmx injection (*Lmna*^*N195K/F:mcm*^ + Tmx) resulted in the cardiomyocyte-specific deletion of the WT floxed *Lmna* allele rendering the CMs hemizygous for the N195K mutation, i.e., *Lmna*^*N195K/–*^. These mice had a mean lifespan of fewer than 50 days, a lifespan half that of the original *Lmna*^*N195K/N195K*^ homozygotes (Supplementary Fig. 2A), whereas *Lmna*^*N195K/+*^ heterozygotes live for more than 1 year. When the *Lmna*^*N195K/F:mcm*^ mutation was induced on a *Sun1* null background, lifespan is significantly extended from <50 days to >200 days (Supplementary Fig. 2A), indicating that loss of *Sun1* is also effective at preventing heart failure caused by *Lmna* missense mutations specifically in CMs.

### Cardiomyocyte-specific ablation of *Lmna* results in impaired heart function

By 21 days post Cre induction, *Lmna*^*F/F:mcm*^ mice showed labored breathing, a disheveled, ungroomed appearance, increased lethargy, and kyphosis (Fig. 2B). PCR and immunofluorescence analysis confirmed the *Lmna* deletion was specific to the *Lmna*^*F/F:mcm*^ CMs, with no detectable recombination occurring in the brain, diaphragm, lung, liver, and skeletal muscle, or in wild-type control animals (Fig. 2C). Lamin A/C protein levels were decreased 3.5 fold in the *Lmna*^*F/F:mcm*^ hearts after Cre induction compared to uninduced *Lmna*^*F/F:mcm*^ and *Lmna*^*+/+/ mcm*^ hearts (Fig. 2D). Immunofluorescence analysis of isolated CMs and sections through *Lmna*^*F/F:mcm*^ hearts revealed reduced levels of Lamin A/C protein with some CM nuclei lacking detectable Lamin A/C (Fig. 2E, F).

**Fig. 3:**
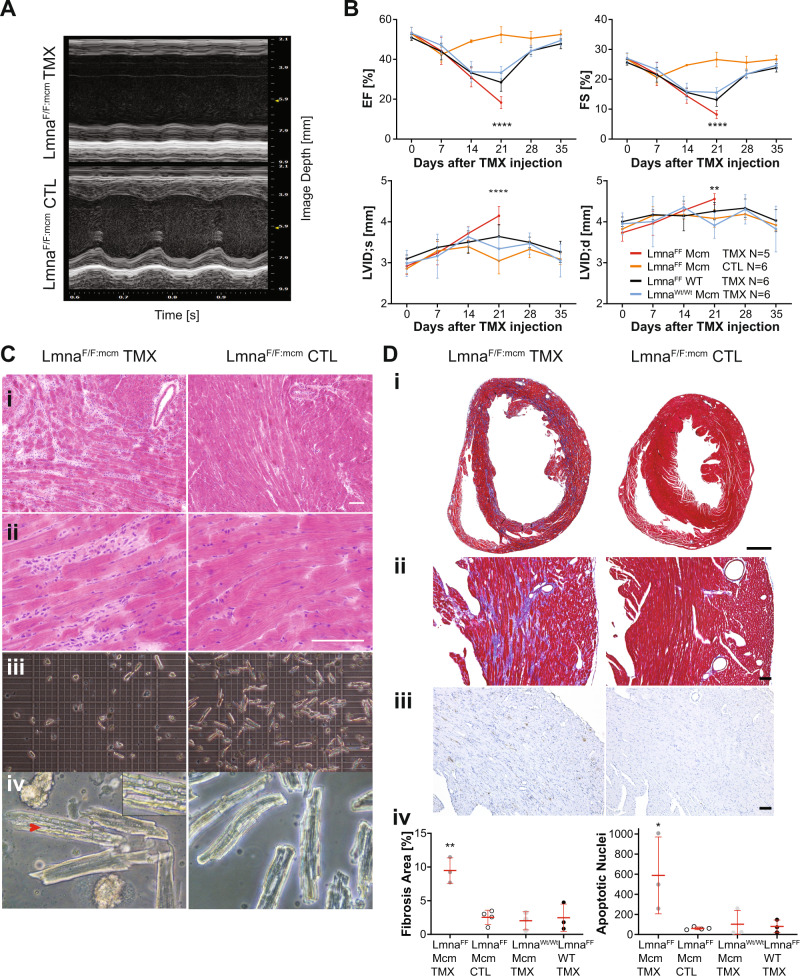
Cardiac function and histology in *Lmna*^*F/F:mcm*^ mice after Cre induction. **A** *Lmna*^*F/F:mcm*^ mice show reduced cardiac contractile function at 21 days after Tmx injection. **B** *Lmna*^*F/F:mcm*^ hearts have reduced EF% and FS%, with increased LVID. Data were analyzed from the total number of animals (*N*) per genotype as indicated in the graph. (****P < 0.0001; **P = 0.0015; one-way ANOVA, mean ± SD). **C** Histological analysis revealed increased nucleated cell infiltration and intercellular spaces in *Lmna*^*F/F:mcm*^ hearts (I and ii). Significantly fewer viable (brick-like) CMs were isolated from *Lmna*^*F/F:mcm*^ hearts compared to *Lmna*^*F/F:mcm*^ controls (*N* = 3 repeats) (iii). The *Lmna*^*F/F:mcm*^ CMs contain large intracellular vacuoles (red arrowhead, iv). Scale bar 100 μm (i), and 50 μm (ii). **D** In *Lmna*^*F/F:mcm*^ hearts the left ventricular lumen was enlarged (i) with increased fibrosis (**P = 0.0007, blue staining) and apoptotic nuclei (*P = 0.0220; one-way ANOVA; mean ± SD) (ii–iv). Scale bar 500 μm (i), 50 μm (ii). All analyses were performed on hearts isolated 21 days after Tmx induction.

Echocardiograms performed 21 days after *Cre* induction revealed poor cardiac contractility in *Lmna*^*F/F:mcm*^ mice compared to *Lmna*^*F/F:mcm*^ controls (Fig. 3A). There was a significant reduction in the Ejection Fraction (EF%) and Fractional shortening (FS%) (*P* < 0.0001) (Fig. 3B). Histological analysis of *Lmna*^*F/F:mcm*^ hearts revealed infiltration of nucleated cells and increased intercellular spaces between CMs compared to *Lmna*^*F/F:mcm*^ control hearts (Fig. 3Ci, Cii). Significantly fewer viable (brick-like) CMs were isolated from the recombined *Lmna*^*F/F:mcm*^ hearts compared to controls (Fig. 3Ciii), with many of the isolated CMs containing large vacuoles (Fig. 3Civ). The left ventricular lumen in Tmx-induced *Lmna*^*F/F:mcm*^ hearts was significantly enlarged (Fig. 3Di). There was an increase in intercellular spaces between *Lmna*^*F/F:mcm*^ + Tmx CMs compared to controls (Fig. 3Dii), together with significantly increased fibrosis (*P* = 0.0098) (Fig. 3Dii). Increased numbers of apoptotic cells (by tunnel analysis) were identified in *Lmna*^*F/F:mcm*^ hearts compared to controls (Fig. 3Diii). However, we did not detect evidence for DNA damage in the CMs, as assessed by Rad51, MRE11, H2AX phosphor-Ser, and 53Bp1 immunostaining.

In the *Lmna*^*N195K/F:mcm*^ mice, echocardiograms after *Cre* induction revealed progressive worsening of cardiac contractility in the *Lmna*^*N195K/F:mcm*^ *Sun1*^*+/+*^ mice compared to *Lmna*^*N195K/F:mcm*^ *Sun1*^*−/−*^ mice (Supplementary Fig. 2B). Loss of SUN1 preserved both EF, FS, and Global Longitudinal Strain (GLS) in *Lmna*^*N195K/–:mcm*^*Sun1*^*−/−*^ mice compared to *Lmna*^*N195K/–:mcm*^ *Sun1*^*+/+*^ mice (Supplementary Fig. 2B).

### Deletion of *Sun1* ameliorates cardiac pathology induced by *Lmna* loss

*Lmna* deletion induced on a *Sun1* null background results in the mice living for more than 1 year after *Cre*-induced deletion of *Lmna* in CMs (Fig. 1B). Hearts and isolated CMs from *Lmna*^*F/F:mcm*^*/Sun1*^*−/−*^ mice, 3 weeks after Tmx induction, were compared to those from *Lmna*^*F/F:mcm*^*/Sun1*^*+/+*^ to determine the extent to which SUN1 loss ameliorated the pathological changes induced by *Lmna* loss in CMs.

**Fig. 4:**
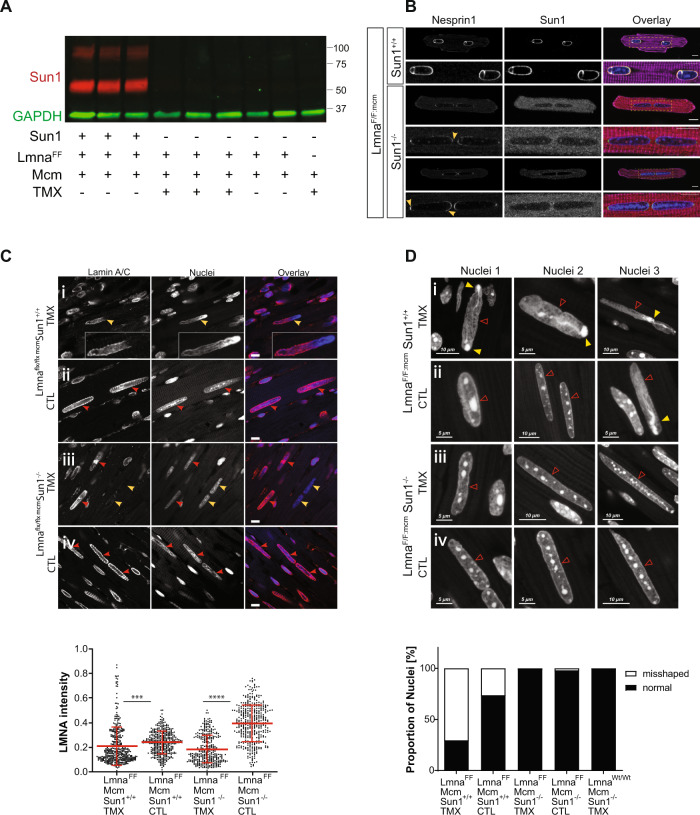
Sun1 loss in *Lmna*^*F/F:mcm*^*/Sun1*^*–/–*^ hearts inhibits changes in nuclear morphology and organization caused by *Lmna* loss. **A** Heart extracts from *Lmna*^*F/F:mcm*^/*Sun1*^*+/+*^ and *Lmna*^*F/F:mcm*^/*Sun1*^*−/−*^ confirmed the absence of Sun1 in the *Sun1*^*−/−*^ hearts (red). GAPDH was used as a loading control (green). A representative blot is shown for the number of animals (*N*) per genotype is indicated below the image. Markers are in kDa (**B**) Immunofluorescence imaging of CMs isolated from 2-month-old *Lmna*^*F/F:mcm*^/*Sun1*^*+/+*^ and *Lmna*^*F/F:mcm*^/*Sun1*^*−/−*^ mice. Absence of Sun1 results in the redistribution of Nesprin1 to the poles of the nuclei indicated by the yellow arrows. Note that the two nuclei in each cardiomyocyte are positioned closer to each other and are elongated. Images are representative for *N* = 3 per genotype shown. Hoechst staining is shown in blue, sarcomeric α-actinin is magenta, (scale bar 10 μm). **C** Immunostaining of CM nuclei in sections through the left heart ventricle 25 days after Cre induction (Tmx). CM nuclei with redistributed or absent Lamin A/C expression are indicated by yellow arrowheads (Ci, Cii). CM nuclei still expressing Lamin A/C are indicated by red arrowheads (Cii, Ciii and iv). Intensity staining and quantification of Lamin A/C levels in nuclei of cardiomyocytes shows a reduction after Cre-induction (***P = 0.0008; ****P < 0.0001; unpaired two-tailed *T*-test, mean ± SD) (lower panel). Data are analysed of nuclei from *Lmna*^*FF:mcm*^*/Sun1*^*+/+*^ Tmx (*n* = 400), *Lmna*^*FF:mcm*^*/Sun1*^*+/+*^ CTL (*n* = 384), *Lmna*^*FF:mcm*^*/Sun1*^*−/−*^ Tmx (*n* = 328) and *Lmna*^*FF:mcm*^*/Sun1*^*−/−*^ CTL (*n* = 399). Scale bar 10 μm. **D** *Lmna*^*F/F:mcm*^/*Sun1*^*+/+*^ CM nuclear morphologies are distorted with indentations (red arrowheads) at the nuclear periphery and DAPI intense foci localizing to the tips of the nuclei (yellow arrowheads). In the absence of Sun1, *Lmna*^*F/F:mcm*^*/Sun*^*−/−*^ CMs show no nuclear indentations or chromatin redistribution. 70% of CM nuclei in *Lmna*^*F/F:mcm*^/*Sun1*^*+/+*^ hearts had NE ruptures/distortions or were misshapen compared to less than 1% of CM nuclei in *Lmna*^*F/F:mcm*^/*Sun*^*−/−*^ Cre-induced mice (lower panel). Data are analysed were of nuclei from 2 animals per genotype *Lmna*^*FF:mcm*^*/Sun1*^*+/+*^ Tmx (*n* = 206), *Lmna*^*FF:mcm*^*/Sun1*^*+/+*^ CTL (*n* = 152), *Lmna*^*FF:mcm*^*/Sun1*^*−/−*^ Tmx (*n* = 131) and *Lmna*^*FF:mcm*^*/Sun1*^*−/−*^ CTL (*n* = 150), *Lmna*^*Wt/Wt*^*/Sun1*^*−/−*^ Tmx (*n* = 144) (No statistical analysis was performed). Source data are provided as a Source Data file.

Western analysis and immunostaining of whole heart extracts revealed the predominant isoform of SUN1 was the Δ10 variant^24^ and confirmed the absence of SUN1 protein (Fig. 4A). In isolated CMs, SUN1 was absent from the periphery of the CM nuclei (Fig. 4B).

Loss of SUN1 resulted in the KASH-domain protein Nesprin-1 localizing to the poles of the CM nuclei instead of being uniformly distributed around the periphery (Fig. 4B). Furthermore, the two nuclei in each CM were both more elongated and had repositioned to be closer to each other in the *Sun1*^*−/−*^ CMs, compared to their broader distribution in *Sun1*^*+/+*^ CMs (Fig. 4B). In Sun1^*−/−*^ cells, a higher background staining is apparent due to increased unspecific binding of the secondary anti-mouse IgG2b antibody.

Immunofluorescence imaging for Lamin A/C on sections from the left ventricles of whole hearts isolated from mice some 3 weeks after *Cre* induction revealed many nuclei to be elongated and distorted. In some of these, residual LaminA was displaced to one pole of the nucleus (Fig. 4Ci and insert) in the *Lmna*^*F/F:mcm*^*/Sun1*^*+/+*^ hearts compared to WT hearts (Fig. 4Cii). In contrast, in the *Lmna*^*F/F:mcm*^*/Sun1*^*−/−*^ hearts, while there were also many elongated nuclei, these showed few if any malformations/indentations, even when there was no Lamin A/C labeling (Fig. 4Ciii yellow arrowheads compared to Fig. 4Civ). Quantification of Lamin A/C intensity on those sections revealed a significant reduction in Lamin A/C protein in the *Lmna*^*F/F:mcm*^*/Sun1*^*−/−*^CM nuclei (***P = 0.0008) compared to *Lmna*^*F/F:mcm*^*/Sun1*^*+/+*^ controls (Fig. 4C lower panels), and comparable to the reduction observed in the *Lmna*^*F/F:mcm*^*/Sun*^+/+^ TMX-induced heart nuclei. The *Lmna*^*F/F:mcm*^*/Sun1*^*+/+*^ CM nuclei exhibited increased longitudinal length, together with a segmented appearance, with the segments often being connected by narrow bridges (Fig. 4D panels i -yellow arrows, compare with ii). In the absence of *Sun1*, *Lmna*^*F/F:mcm*^*/Sun1*^*−/−*^ CM nuclei exhibited no chromatin abnormalities or segmentation defects (Fig. 4Diii and iv). In total, 70% of CMs in *Lmna*^*F/F:mcm*^*/Sun1*^*+/+*^ mice had ruptured or misshapen nuclei compared to fewer than 1% of the CMs from the *Lmna*^*F/F:mcm*^*/Sun1*^*−/−*^ animals (Fig. 4D lowest panel).

**Fig. 5:**
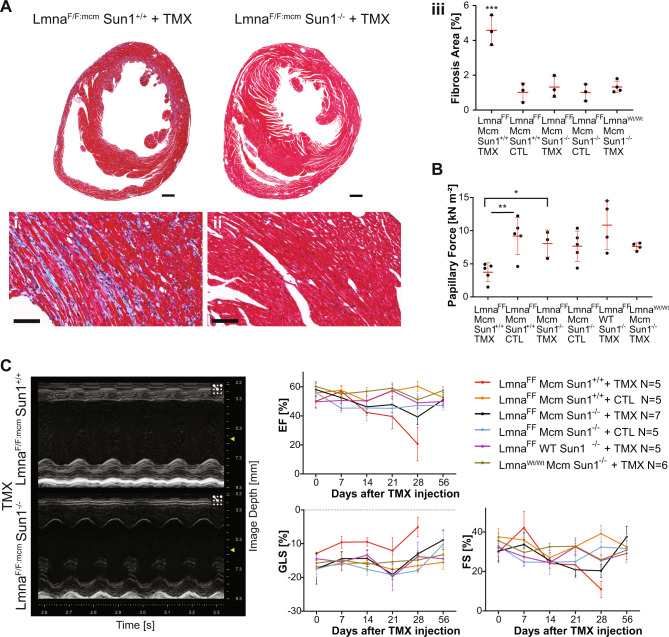
Cardiac pathology of *Lmna*^*F/F:mcm*^*/Sun1*^*−/−*^ hearts after Cre induction. **A** Left ventricular enlargement (LV) was apparent in the Cre-induced (TMX) *Lmna*^*F/F:mcm*^/*Sun1*^*+/+*^ (*n* = 3) hearts but not in the LV of the Cre-induced (TMX) *Lmna*^*F/F:mcm*^/*Sun1*^*−/−*^ (*n* = 3) hearts. The *Lmna*^*F/F:mcm*^/*Sun1*^*+/+*^ induced (TMX) hearts had significantly increased fibrosis (***P = 0.0001; One-way ANOVA), compared to controls, with no significant fibrosis in the *Lmna*^*F/F:mcm*^*/Sun1*^*−/−*^ hearts compared to controls (i–iii). The following genotypes served as controls: *Lmna*^*FF:mcm*^*/Sun*^*+/+*^ (CTL) (*n* = 3), *Lmna*^*FF:mcm*^*Sun1*^*−/−*^ (CTL) (*n* = 3), and *Lmna*^*Wt/Wt:mcm*^*/Sun1*^*−/−*^ induced (TMX) (*n* = 4). bar 500 μm, 100 μm (i + ii). **B** Cardiac papillary muscle active force measurements were significantly reduced from the Cre-induced (TMX) *Lmna*^*F/F:mcm*^/*Sun1*^*+/+*^ mice (*n* = 5) compared to *Lmna*^*F/F:mcm*^/*Sun1*^*+/+*^ controls (CTL) (*n* = 5) (unpaired two-tailed *T*-test) and Cre-induced (TMX) *Lmna*^*F/F:mcm*^/*Sun1*^*−/−*^ (*n* = 3) (**P = 0.0047, *P = 0.0113; unpaired two-tailed *T*-test). The following genotypes served as controls: *Lmna*^*FF:mcm*^*/Sun1*^*−/−*^ (CTL) (*n* = 5), *Lmna*^*FF*^*Sun1*^*−/−*^ induced (TMX) (*n* = 4), and *Lmna*^*Wt/Wt:mcm*^*/Sun1*^*−/−*^ induced (TMX) (*n* = 4). Quantification was performed based on five fibers per animals. **A**, **B** Data are presented as mean ± SD. **C** ECGs were performed up to 28 days after Tmx induction on 3–5-month-old mice. ECGs performed before and after *Cre* induction revealed a progressive worsening of cardiac contractility in *Lmna*^*F/F:mcm*^/*Sun1*^*+/+*^ Cre-induced mice (red line) compared to *Lmna*^*F/F:mcm*^/*Sun1*^*−/−*^ mice. The loss of Sun1 preserved both EF, FS, and Global Longitudinal Strain (GLS) in *Lmna*^*F/F:mcm*^/*Sun1*^*−/−*^ hearts compared to *Lmna*^*F/F:mcm*^/*Sun1*^*+/+*^ hearts. Echo images were recorded at 28 days after Tmx induction. Data were analysed from the total number of animals per genotype (*N*) as indicated in the graph. Data are presented as mean ± SD.

At the tissue level, enlargement of the left ventricle (LV) was evident in the *Lmna*^*F/F:mcm*^*/Sun1*^*+/+*^ mice but not in the lives of the *Lmna*^*F/F:mcm*^*/Sun1*^*−/−*^ hearts (Fig. 5A). The *Lmna*^*F/F:mcm*^*/Sun1*^*+/+*^ hearts exhibited significantly increased fibrosis (***P < 0.0001) compared to controls, whereas slight fibrosis was evident in the *Lmna*^*F/F:mcm*^*/Sun1*^*−/−*^ hearts (Fig. 5A lower panels and graph).

To assess ventricular muscle mechanics, we measured the active force in cardiac papillary muscle. The active force was reduced by 66% in *Lmna*^*F/F:mcm*^*/Sun1*^*+/+*^*+*Tmx papillary muscle (*P* = 0.0028) when compared with *Lmna*^*F/F:mcm*^*/Sun1*^*+/+*^ controls. In the absence of SUN1, *Lmna*^*F/F:mcm*^*/Sun1*^*−/−*^ + Tmx cardiac papillary active force was maintained at levels that did not differ significantly from those of controls (Fig. 5B).

Echocardiograms performed before and after *Cre* induction revealed a progressive worsening of cardiac contractility in the *Lmna*^*F/F:mcm*^*/Sun1*^*+/+*^ hearts compared to *Lmna*^*F/F:mcm*^*/Sun1*^*−/−*^ hearts (Fig. 5C). The absence of SUN1 preserved both EF, FS and GLS (GLS is a separate parameter used to assess myocardial contractility, and is a better predictor of heart failure) in the *Lmna*^*F/F:mcm*^*/Sun1*^*−/−*^mice compared to *Lmna*^*F/F:mcm*^*/Sun1*^*+/+*^ mice.

PCR analysis of *Lmna*^*F/F:mcm*^*/Sun1*^*−/−*^ hearts 12–14 months after Cre induction confirmed the sustained deletion of the *Lmna* gene (Supplementary Fig. 3A), and protein quantification revealed a significant reduction of Lamin A/C levels in these hearts (Supplementary Fig. 3B). Histological analysis of the hearts revealed no significant increase in fibrosis than controls (Supplementary Fig. 3C, D). However, echocardiograms showed reduced EF and FS in both the *Lmna*^*F/F:mcm*^*/Sun1*^*+/+*^ (uninduced) and *Lmna*^*F/F:mcm*^*/Sun1*^*−/−*^ +Tmx mice (Supplementary Fig. 3E). The average lifespan of *Lmna*^*F/F*^ mice is 13–14 months, implying that the loxP sites may compromise lamin expression, resulting in reduced contractile function as the mice aged. Together these findings demonstrate that loss of *Lmna*, in 2–3 month adult CMs is sufficient to result in cardiac failure within 3–4 weeks after *Cre* activation. However, the pathology is significantly reduced by deleting *Sun1*, resulting in cardiomyopathy progression being retarded for at least a year.

### AAV9 mediated transduction of a DNSUN1 ameliorates cardiomyopathy

**Fig. 6:**
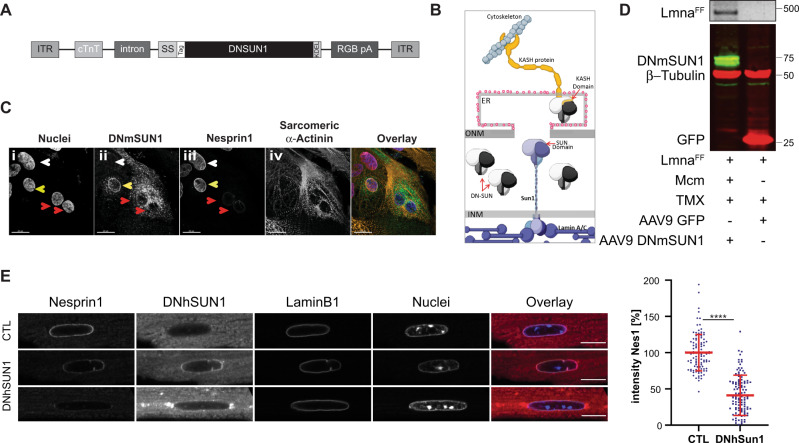
Expression of the DNSUN1 miniprotein reduces Nesprin1 localization at the nuclear envelope in cardiomyocytes. **A** The AAV9 human/murine SUN1 dominant-negative construct (AAV9-DNhSUN1). The dominant-negative SUN1 sequence encompasses parts of the luminal domain, including the SUN domain. To detect the transgene in tissues, either an HA-tag or a MYC-tag sequence was fused on the NH2 terminus of the murine and human DNSUN1, respectively. ITR = AAV2 inverted terminal repeat; cTnT = Chicken cardiac troponin promotor; intron = β-globin/IgG chimeric intron; Signal sequence = 1–25aa of human serum albumin (Uniprot P02768 signal peptide + propeptide); Tag = HA epitope tag used with the murine DNmSUN1; Myc epitope tag used with human DNhSUN1; SUN1DN = 1046–2404 nt of NM_001130965; KDEL = Golgi-to-ER retrieval sequence; RGB pA = Rabbit globin polyA tail. **B** The DNmSUN1 miniprotein competes with endogenous SUN1 for binding to the KASH-domain of the Nesprins (which in CMs is Nesprin1). It displaces Nesprin1 from the nuclear envelope, disrupting LINC complex-mediated attachment of the nucleus to the cytoskeleton. **C** Induced pluripotential stem cell derived CMs were transduced with the AAV AJ-D to express DNmSUN1. The DNmSUN1 miniprotein is not anchored in the INM and so competes with endogenous SUN protein complexes from binding to the KASH domains of Nesprin1 in CMs, so breaking the LINC complex as outlined in (**B**). It displaces the Nesprin1 from the NEs of IPS-derived cardiomyocytes (Cii and iii). CMs expressing the transduced DNmSUN1 are indicated by the red arrows and show loss of Nesprin1 from the nuclear envelope (NE) (red arrows). In contrast, in CMs not expressing DNmSUN1 or at lower levels, Nesprin1 still localizes to the NE (yellow and white arrows- panel iii). The image shown is representative of 3 independently performed experiments. **D** The recombined *Lmna* gene following Cre induction was confirmed by PCR of the heart tissues (upper panel). Robust expression of both AAV9-DNmSun1 and AAV9-GFP protein (Dosage: 5 × 10^10^ vg/g per mouse) was detected in extracts from whole hearts 99 days post AAV injection (lower panel). Western blot was repeated twice with *N* = 4 per AAV9 construct. Markers in kDA. **E** Isolated CMs from mice transduced with AAV9 delivering DNhSUN1 (DNhSUN1) or controls injected with PBS only (CTL). CMs were immunostained with anti-Nesprin1 and anti-hSUN1. The intensity or levels of Nesprin1 at the NE were reduced by the expressed DNhSUN1 revealing displacement of Nesprin1 protein (scale bar 10 μm). Nesprin1 signal intensity at the NE was quantified and showed a drop in the median levels of Nesprin1 intensity at the nuclear rim to 41.13% in CMs isolated from AAV9-DNhSUN1 transduced mice (****P < 0.0001, unpaired two-tailed *T*-test, mean ± SD). *n* = 35 over three independent experiments. Source data are provided as a Source Data file.

These results demonstrate that genetic ablation of SUN1 functionality or reduced possibly toxic SUN1 levels could be of therapeutic value in treating DCM. Consequently, our next goal was to determine whether the beneficiary effects of SUN1 loss were due to the complete elimination of SUN1’s functions, including interactions involving its nucleoplasmic domain, versus specific disruption of its LINC complex-associated role. In the latter, SUN1 functions as an anchor for KASH-domain proteins in the ONM, and mediate the tethering of the nucleus to components of the cytoskeleton, with the microtubular network, preferentially interacting with SUN1-KASH LINC complexes^35^. To distinguish between these two possibilities, we utilized the Adenovirus Associated Virus (AAV) to transduce and specifically express in CMs, a dominant-negative SUN1 minigene whose protein product would compete with SUN1-KASH binding in the CM perinuclear space^22^ (Fig. 6A). Initially, we used a region corresponding to the entire luminal domain of the mouse *Sun1* gene, which was tagged at its N terminus with an HA (HA-SUN1L) epitope. To localize the resulting protein product to both the lumen of the endoplasmic reticulum (ER) and perinuclear space (between the INM and ONM-PNS), the signal sequence, and signal peptidase cleavage site of human serum albumin was fused to the N terminus of HA-Sun1L to yield SS–HA-SUN1L. To prevent the secretion of the miniprotein from the ER-PNS, a KDEL tetrapeptide was linked to the C- terminus of SS–HA-Sun1L, forming SS–HA-SUN1L–KDEL^22^ (Fig. 6A). The signal sequence would ensure the HA-SUN1L-KDEL accumulates intracellularly within the contiguous peripheral ER and PNS lumen. The cDNA sequence encoding the minigene was fused to the chicken cardiac troponin promoter (cTnT) to ensure the minigene is only transcribed in CMs^36^. A diagram of how SS–HA-Sun1L (DNSUN1) competes with endogenous SUN1, thereby displacing KASH-domain proteins from NE-associated LINC complexes, is presented in Fig. 6B.

To verify that the DNmSUN1 functioned in CMs, we initially transduced human CMs derived from iPS stem cells using the AVV-DJ system to provide a higher infectivity rate in cultured cells than the AAV9 serotype (Fig. 6C). The DNmSUN1, under transcriptional control of the cTnT promoter, effectively displaced Nesprin1 from nuclear envelopes in the CMs expressing the DNmSUN1 (Fig. 6Cii and iii) confirming that the DNmSUN1 could disrupt LINC complexes in CMs.

We then used AAV (serotype 9) to transduce and express the DNmSUN1 minigene in the hearts of postnatal mice, initially by intrathoracic injection at a dose of 5 × 10^10^ vg/g per mouse. The injected mice were sacrificed for analysis at 99 days after Tmx-induced deletion of *Lmna*. Detection by PCR of the *Lmna* deletion in the hearts confirmed *Cre* induction following Tmx injection as expected (Fig. 6D). The localization and expression levels of the DNmSUN1 minigene was determined by total protein extraction from half the heart. Western analysis revealed robust expression of both AAV9-DNmSUN1 and AAV9-GFP control protein 99 days after AAV injection (Fig. 6D lower panel), with the expression levels of both proteins being dependent on the dose of viral particles injected (Supplementary Fig. 4B, C).

To further verify that the DNmSUN1 was disrupting the LINC complex, displacing Nesprin1 in CMs, CMs were isolated from mice transduced with AAV9 delivering DNmSUN1 or controls injected with PBS only. The CMs were immunostained with anti-Nesprin1 and anti-SUN1. The intensity or levels of Nesprin1 at the NE were reduced, revealing displacement of Nesprin1 protein. (Fig. 6E). Nesprin1 signal intensity at the NE was quantified and revealed a drop in the median of Nesprin1 intensity at the nuclear rim to 41.13% in CMs isolated from AAV9 DN-mSUN1 transduced mice (*t* test ****P < 0.0001).

In these experiments, *Lmna*^*F/F:mcm*^ mice injected with the AAV9-GFP control lived an average of 34.5 days after Cre induction (Supplementary Fig. 5A). In contrast, *Lmna*^*F/F:mcm*^ mice injected with AAV9-DNmSUN1 (5 × 10^10^ vg/g) lived significantly longer, with the majority surviving at least 99 days after Tmx induction, before being sacrificed for analysis (*P* = 0.0002) (Supplementary Fig. 5A). At 35 days post-Tmx injection, fibrosis was detected in both the *Lmna*^*F/F:mcm*^ + AAV9-DNmSUN1 and *Lmna*^*F/F:mcm*^ + AAV9-GFP hearts (Supplementary Fig. 5B), although fibrosis levels in the *Lmna*^*F/F:mcm*^ + AAV9-DNmSUN1 hearts was significantly lower than the levels in the *Lmna*^*F/F:mcm*^ + AAV9-GFP hearts (Supplementary Fig. 5B lower panels). ECG analysis confirmed *Lmna*^*F/F:mcm*^ + AAV9-DNmSUN1 hearts were functioning better than *Lmna*^*F/F:mcm*^ + AAV9-GFP hearts at 35 days post-Tmx (Supplementary Fig. 5C). Although the *Lmna*^*F/F:mcm*^ + AAV9-DNmSUN1 mice were alive at 100 days after induction, both EF% and FS% were lower compared to control *Lmna*^*F/F:Wt*^ mice (Supplementary Fig. 5C). The expression of either the AAV9-DNmSUN1 or AAV9-GFP proteins did not affect Lamin A/C protein levels (Supplementary Fig. 4A). Varying doses of either AAV9-DNmSUN1 or AAV9-GFP were injected into mice to analyze the correlation of concentration of virus administered to the transgene expression by western blot analysis of whole heart lysates after 35 days. This revealed GFP expression, and the DNmSUN1 miniprotein are expressed in a dose-dependent manner (Supplementary Fig. 4B). Immunofluorescence analysis revealed GFP expression in the transduced hearts was more intense following injection with 5 × 10^10^ vg/g of AAV9-GFP than expression levels from a 10-fold lower dose of (5 × 10^9^ AAV9-GFP) (Supplementary Fig. 4C). Expression was almost entirely restricted to the heart, with minimal expression detected in the liver and only after injection at the highest concentrations (Supplementary Fig. 4D).

**Fig. 7:**
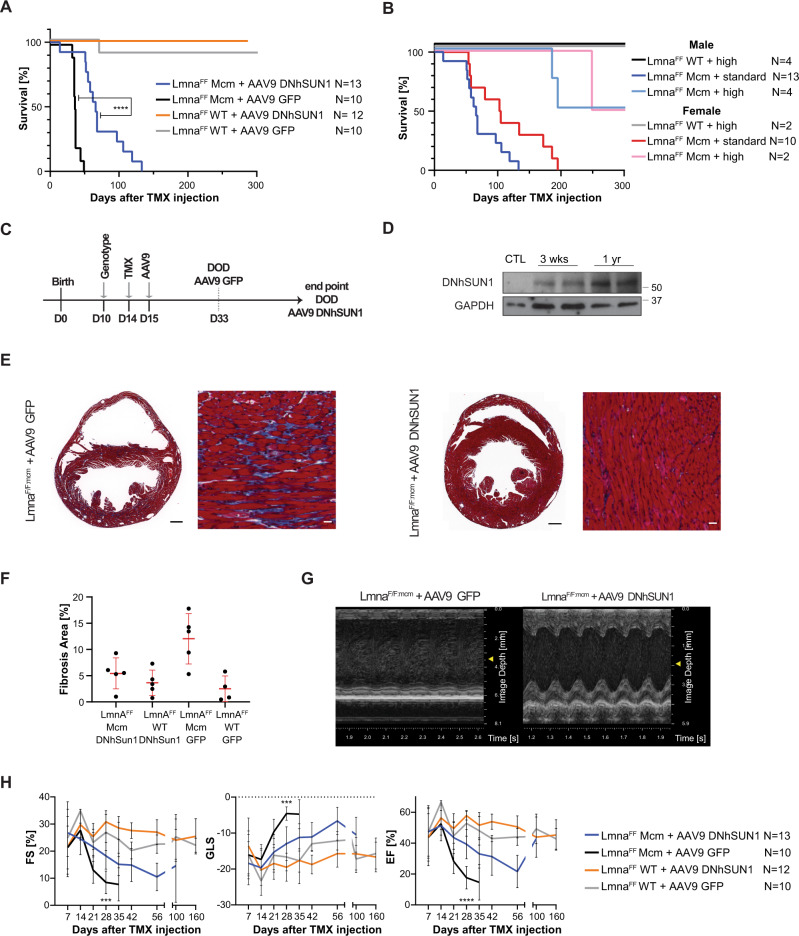
*Lmna*^*F/F:mcm*^ mice transduced with AAV9-DNhSUN1 show improved cardiac function and extended longevity. **A** Transduction of AAV9-DNhSUN1 extends the lifespan of the *Lmna*^*F/F:mcm*^ mice after tamoxifen induction. *Lmna*^*F/F:mcm*^ + AAV9-DNhSUN1 transduced mice with the standard dose (2 × 10^10^ vg g^−1^) live for a median of 66 days post-Tmx induction, whereas the *Lmna*^*F/F:mcm*^ + AAV9-GFP transduced mice have a shorter lifespan (36.5 days) (*P* < 0.0001; Log-rank (Mantel–Cox) test). All animals were induced with Tmx. **B** Transduction of a double dose (4 × 10^10^ VG/g) of AAV9 DNhSUN1 increases the lifespan of *Lmna*^*F/F:mcm*^ animals. Male *Lmna*^*F/F:mcm*^ injected with a double dose of DNhSUN1 live for an average of 205 days and females to 309 days post-Tmx induction, whereas the *Lmna*^*F/F:mcm*^ injected with the standard dose DNhSUN1 have a shorter median lifespan (males 66 days, females 104 days). **C** Experimental procedure for AAV transduction. Tmx induction by IP injection at postnatal day 14, followed on day 15 in initial experiments by intrathoracic injection of AAV9-DNmSUN1 or AAV9-GFP as control (AAV). In subsequent experiments with the DNhSUN1 delivery was by retro-orbital injection. The expected lifespan of *Lmna*^*F/F:mcm*^ injected with AAV9-GFP was 33 days after Tmx induction. The endpoint of this study is the date of death of *Lmna*^*F/F:mcm*^ animals injected with AAV9-DNhSun1 (DOD). **D** The DNhSUN1 protein is detected by western blot analysis at 3 weeks after injection and after 1 year following injection at similar levels in whole heart lysates with an antibody specific to the C-terminus of human SUN1. A representative blot is shown for the number of animals (*N* = 2) per condition. Western blots were performed in triplicate. Markers in kDA (**E** + **F**) At 21 days after Tmx induction, extensive fibrosis (blue staining) was detected in *Lmna*^*F/F:mcm*^ mice injected with AAV9-GFP (*N* = 5) in comparison to *Lmna*^*F/F:mcm*^ animals treated with AAV9-DNhSUN1 (*N* = 5). The following genotypes served as controls: *Lmna*^*F/F*^ + AAV9-DNhSUN1 (*N* = 5), *Lmna*^*F/F*^ + AAV9-GFP (*N* = 4). Data are presented as mean ± SD. (scale bars 500 μm, 40 μm). **G** ECG analysis of mice injected with a standard AAV-DNhSUN1 dose shows improved cardiac function at day 28 after Tmx induction. **H** Cardiac function after transduction with AAV9-DNhSUN1 is improved in *Lmna*^*F/F:mcm*^ animals. ECG analysis revealed an significant improvement of Fractional Shortening (FS), Global Longitudinal Strain (GLS) and Ejection Fraction in *Lmna*^*F/F:mcm*^ + AAV9-DNhSun1 animals compared to *Lmna*^*F/F:mcm*^ + AAV9-GFP control animals at day 28 (FS ***p = 0.0009; GLS ***p = 0.0003; EF ****p < 0.0001; One-way ANOVA with Tukey correction). Data were analyzed from the total number of animals (N) per genotype as indicated in the graph. Data are presented as mean ± SD. Source data are provided as a Source Data file.

A subsequent, more extensive series of AAV transductions were performed using a human DNhSUN1 construct. Instead of using the intrathoracic route for introducing the AAV suspension, we used retro-orbital injection^37^. *Lmna*^*F/F:mcm*^ mice injected with the AAV9-GFP control lived an average of 34.5 days after Cre induction (Fig. 7A). In contrast, *Lmna*^*F/F:mcm*^ mice injected with AAV9-DNhSUN1 (2 × 10^10^ VG/g) lived significantly longer, with the majority surviving at least 66 days after Tmx induction before they sacrifice for analysis (*P* = 0.0002) (Fig. 7A). In Fig. 7B, the results of both low (2 × 10^10^ vg/g) and high (4 × 10^10^ VG/g) doses of DNhSUN1 are presented and show that doubling the concentration of the injected DNhSUN1 extends longevity even further to at least 300 days after Tmx injection. The results further suggested that females may survive longer than males; a divergence also noted in mice where *Lmna* deletion in the liver^38^. Injection of the DNhSUN1 into wild-type mice revealed the DNhSUN1 had no overt detrimental effects on the longevity of normal mice with the mice living for over 1 year with DNhSUN1 protein expression still being detectable in heart extracts after 1 year (Fig. 7D). Histological examination of the hearts revealed significantly reduced fibrosis in the DNhSUN1 expressing hearts (Fig. 7E, F) with conserved ventricular dilation in the DNhSUN1 transduced hearts (Supplementary Fig. 6A, D). ECG analysis confirmed *Lmna*^*F/F:mcm*^ + AAV9-DNhSUN1 hearts were functioning better than *Lmna*^*F/F:mcm*^ + AAV9-GFP hearts at 28 days post-Tmx (Fig. 7G, H), with heart function, also improved by doubling the dose of AAV (Supplementary Fig. 6C, E). Although the *Lmna*^*F/F:mcm*^ + AAV9-DNhSUN1 mice were alive at 100 days after induction both EF% and FS% were still lower compared to control *Lmna*^*F/F:Wt*^ mice (Fig. 7H). Importantly, expression of the DNhSUN1 in Wt mice had no detrimental effect on cardiac function at 160 days after Tmx.

## Discussion

Here we show that disrupting the LINC complex protein, SUN1, suppresses DCM progression caused by *LMNA* mutations. *LMNA* associated DCM is regarded as a particularly aggressive form of heart failure, frequently leading to premature death or cardiac transplantation^39,40^. By 60 years, 55% of *LMNA* mutation patients die of cardiovascular failure or receive a heart transplant, compared with 11% with idiopathic cardiomyopathy. Attempts to ameliorate DCM by fitting a pacemaker have been, at best, of transient benefit. Consequently, it is necessary to develop new therapeutic avenues to treat DCM caused by *LMNA* mutations.

The majority of *LMNA* mutations causing DCM are dominant missense, primarily due to a single base change. Treatment by gene therapy to repair each mutation would be a daunting task. Moreover, simply eliminating the mutated allele, leaving the patient haplo-insufficient for the remaining WT allele would almost certainly be ineffective at preventing heart failure as a patient, who was effectively heterozygous for *LMNA*, developed DCM^41^. Various other routes downstream of the *LMNA* gene have been explored as potential therapeutic pathways. These have included inhibiting mTOR with rapamycin/rapalogues^42,43^, MEK1/2 kinase pathway inhibitors^44^, upregulation of YY1^45^, and most recently inhibiting the transcription factor bromodomain-containing protein 4 (BRD4)^46^. Many of these procedures necessitated repeated injections (often daily) of the compounds, some of which were associated with significant side effects.

In contrast, the AAV delivery system requires a single injection. Our data revealed that following a single injection of the vector expression of the DNSUN1 constructs is still detectable 1 year after injection. In all studies, as here, the primary endpoint was lifespan extension, with fibrosis reduction and cardiac function being the secondary endpoints. All approaches resulted in increased longevity, improved ventricular function, and reduced fibrosis (10–40%), but the lifespan extension and long-term efficacy were less than that observed by depletion and genetic disruption of SUN1. However, longevity is extended to close to 1 year after administering and persistent expression of the DNhSUN1 protein for at least 1 year.

The molecular mechanisms underlying the varied phenotypes of the laminopathies are still not understood, though two alternative hypotheses have been proposed to explain the tissue-specific pathologies^28^. The first “gene regulation hypothesis” proposes that *LMNA* mutations/loss disrupt the equilibrium of various molecular pathways due to the mutations altering interactions with NE proteins and chromatin, altering gene expression. Evidence supporting this hypothesis comes from studies reporting changes in signaling pathways including the AKT-mTOR^42^, WNT/β-catenin^47,48^, TGF-β/Smad^5,19^, MAP Kinase^49^ and the ERK1/2–CTGF/CCN2 pathways^50^. While all these changes have been documented, it is not established whether these changes are merely a secondary compensatory effect in diseased tissue.

The second hypothesis centers on *LMNA* loss or mutation *that* leads to increased nuclear fragility. As a result, mechanical stress and tension forces transmitted via the LINC complex from the cytoplasm to the NE causes damage to the NE^51^. This hypothesis is similar to that proposed for Duchenne muscular dystrophy (DMD), where the loss of dystrophin increases the fragility of the muscle cell membrane, making them susceptible to tension-stress forces during muscle contraction and results in muscle cell rupture and death^52^. *LMNA* mutant fibroblasts show nuclear deformation, defective mechanotransduction, reduced viability when subjected to mechanical strain, and increased nuclear rupture at low and moderate pressures compared to WT nuclei^51,53,54^. Within the context of contracting murine CMs, mechanical stress and tension forces caused by 500–600 contractions per minute are exerted on the NE via the LINC complex, resulting in nuclear distortion, damage, and eventual death/loss as presented in Figs. 3, 4. Such forces may cause significant damage to the fragile NE of *Lmna*-null CMs, resulting in CM death. Whether such forces also induce DNA damage in the CMs, as reported for myoblasts derived from *Lmna* mutant mice, is still unclear^55^ as we could not detect any convincing evidence for DNA damage in the LaminA depleted CMs. If this tension-stress hypothesis is correct and that the cytoskeleton itself promotes damage to the NE, then unlinking the LINC complex by interfering with SUN1 function should reduce the stress on the CM nuclei. Such uncoupling would predict the prevention of CM cell death in the mutant CMs. To test this tension-stress hypothesis, a DNhSUN1 construct^22^ was used to compete with endogenous SUN1 for KASH-domain-binding to decouple CM LINC complexes (Fig. 6B, E). The AAV9 vector, which has a high affinity for CMs, was used to deliver the DNSUN1 hybrid gene under the control of the cTnT promoter to CMs^56^. Our results showed the successful delivery and expression of the GFP tagged miniprotein to CMs (Supplementary Fig. 4C), with robust expression of both the control GFP and DNSUN1 proteins (Figs. 6, 7D) with expression persisting 1 year after injection, indicating significant perdurance of the DNSUN1 protein (Fig. 7D). Minimal off-target expression, i.e., liver, was detected only after injection of very high vector concentrations (Supplementary Fig. 4D). Significantly, expression of DNSUN1 was associated with reduced levels of KASH-domain protein (Nesprin1) from the CM NEs (Fig. 6C, E).

**Fig. 8:**
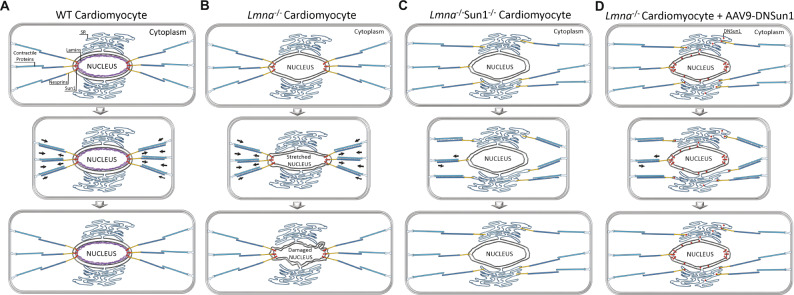
Breaking the LINC by disrupting Sun1 protects cardiomyocytes from contraction-induced stress. **A** CM nuclei expressing normal *Lmna*, withstand mechanical stress and tension forces transmitted via the LINC complex from the sarcoplasmic cytoskeleton, predominantly the MT system to the NE. **B** The loss of or introduction of a mutation within the *Lmna* gene results in loss/or incorrect assembly of the nuclear lamina, resulting in a compromised and weakened Lamina/NE. The weakened nuclei are damaged due to the tension/stress forces exerted via the MT network and LINC complex from the contractile sarcomeres of the cardiomyocytes. **C**, **D** In the absence of SUN1 or by disrupting its binding to the KASH domains by expression of DNSUN1, the now untethered LINC complexes exert less tensional force on the CM nuclei, enabling the survival of the *Lmna* mutant CMs.

These results indicate that loss or mutation of *LMNA* results in instability of the CM lamina and nuclei due to incorrect assembly of the lamins. Incorrect lamin assembly, therefore, makes the nuclei susceptible to the tension/stress forces exerted via the LINC complex from the contractile sarcomeres of the CMs or other force-generating/transmitting cytoskeletal components (Fig. 8A, B). In the absence of functional SUN1, due to *Sun1* gene ablation or DNSUN1 expression disrupting the SUN1 trimer complex^57^, tethering of the KASH proteins to the ONM is decreased. The KASH domain or at least Nesprin-1 association with the NE is then diminished, resulting in reduced tensional force transmission to the CM nuclei, thereby enabling the survival of CMs with an otherwise compromised lamina (Figs. 8C, 7D). Intriguingly, the loss of SUN2, the other widely expressed SUN domain protein, did not rescue the mice carrying *Lmna* mutations. In mice, SUN2 loss induces mild cardiac hypertrophy^24^, and although SUN1 and SUN2 show genetic redundancy during embryogenesis, SUN1 preferentially interacts with the cytoplasmic microtubular (MT) networks, whereas SUN2 preferentially interacts with the cytoplasmic microfilament-actin network^35^. In addition, SUN1 interacts directly with Lamin A whereas SUN2 does not or shows reduced interaction^58^. In CMs, the MTs ramify throughout the cell, but cluster as a dense network around the CM nuclei and, therefore, may be the principal cytoskeletal network the LINC complex/Nesprin-1/SUN1 interacts.

In contrast, actin microfilament networks i.e., stress fibers or transmembrane TAN lines, are absent, as actin predominantly localizes to the sarcomeres^59^. Intermediate filaments, in particular Desmin, and its interaction with the KASH-domain protein Nesprin 3, has also been implicated in maintaining CM nuclear organization. However, in our hands, Nesprin3 null mice are overtly normal, and we did not detect any cardiovascular defect either histologically or by changes in blood pressure. These findings suggest that disruption of the SUN1/Nesprin1 LINC complex specifically uncouples the CM nuclei from mechanical stresses transmitted through the CM MT network^60^.

Our results provide an opportunity to use the AAV-DNSUN as a potential therapeutic for preventing DCM progression in laminopathy patients. An attractive feature to using the DNSUN1 as a therapeutic is its potential long perdurance, which may avoid the necessity for repeat AAV injections, resulting in immune responses to the AAV, potentially compromising the efficacy of the vector. As a delivery route for patients, the AAV system is established and approved by regulatory authorities to treat an increasing number of diseases. It is becoming more widely used with multiple ongoing clinical trials, including its introduction into patients with heart disease^61,62^. However, even though tension-stress may be the primary cause for *LMNA* deficient CM death, disrupting SUN1 may not be so effective in preventing *Lmna* mutation-induced cell death in murine skeletal muscle, as *Lmna*^*Δ/Δ*^:*Sun*^*–/–*^ mice die at an earlier age than those mice where *Lmna* was specifically deleted in the CMs. Which muscle groups (or even other tissues lacking *Lmna*) result in the earlier murine lethality remain to be identified. However, in the *LMNA* DCM patients, heart failure is the primary cause of death, and our results show that disrupting the LINC complex in CMs could be effective at retarding heart failure.

## Methods

### Mouse lines

Mouse (C57Bl6/J and 129 Sv/J) strains were maintained at the A*STAR Biological Resource Center facility and the NUS Animal Facility on a 12 h light/dark cycle in ventilated animal barrier facilities with the temperature set to 21 ± 1 °C, humidity at 55–70% and with food and water provided *ad libitum*. Ethical oversight and approval were granted by the Institutional Animal Care and Use Committees, for both the ASTAR Biological Resource Center (BRC) and the NUS AICUC and the animal facility/committee (Comparative Medicine protocol R16-213) and is governed by the association of AAALAC (USA) providing guidelines to both AALAS (USA) and AVS (Singapore) to which NUS adheres.

The *Lmna*^*F/F*^ mice were generated and characterized as previously described^14,63^. To derive mice with a global deletion *Lmna* (*Lmna*^*∆/∆*^), we crossed the floxed allele (*Lmna*^*F/F*^) to mice in which Cre recombinase is driven by the regulatory sequences of the mouse zone pellucida 3 gene (Zp3;Tg(Zp3-cre)93Knw, JAX stock 003651)^30^. Cardiomyocyte (CM)-specific deletion of *Lmna* (*Lmna*^*F/F αIMhc*^), was performed using 2 lines where *Cre* recombinase is expressed specifically in CMs. We first crossed the *Lmna*^*F/F*^ mice to mice where Cre expression was driven by the CM-specific murine alpha myosin-heavy chain (Myh6, myosin, heavy polypeptide 6, cardiac muscle, alpha) promoter (MyHC;Tg(Myhca-cre)2182Mds, JAX stock 011038). In addition, we derived a tamoxifen-inducible CM-specific deletion of *Lmn*a (*Lmna*^*F/F:mcm*^), by crossing the *Lmna*^*F/F*^ with a line where Cre expression was driven by the mouse CM-specific alpha-myosin heavy chain promoter (αMHC or alpha-MHC;Myh6 abbreviated to *mcm*) that expresses a tamoxifen-inducible Cre recombinase (MerCreMer) specifically in juvenile (14d) and adult (3–36 month) cardiac myocytes (mcm;Tg(Myh6-cre/Esr1*)1Jmk, JAX stock 005657). The specificity of *mcm* Cre expression to CMs was confirmed by crossing *Cre* lines to the mT/mG reporter mice^64^. Generation of the *Sun1*^*−/−*^mice was previously described^65^, as was the *Lmna*^*N195K/N195K*^^12^ line that carries a *Lmna* N195K missense mutation that also results in death associated with heart failure. The *Lmna*^*∆/∆*^*:Sun1*^*−/−*^ and *Lmna*^*F/F mcm:Sun1−/−*^ mice were obtained by crossing the respective Lamin-Cre mice strains with *Sun1*^*+/-*^ mice as *Sun1*^*−/−*^ mice are infertile.

To test for the insertion of loxP sites and the conditional deleted allele, genotyping was performed with a duplex PCR protocol. The primer sequences are in Supplementary Table 1.

### Tamoxifen injection and tissue collection

Mice (14 days old) and adults (3–5 months old) were injected once with 40 mg/kg of Tamoxifen (Sigma) dissolved in corn oil (Sigma). Mice were sacrificed by CO_2_ euthanasia or anaesthetized with a gaseous mixture of 1.5% Isoflurane (BioMac) and 1.5 L O_2_ at various time points after tamoxifen injection. Cardiac arrest was induced by injection of 15% KCl, followed by flushing with PBS to remove blood. Hearts for paraffin embedding were flushed with 4% paraformaldehyde (PFA), left in 4% paraformaldehyde (PFA) overnight, dehydrated in 70% ethanol for at least 24 h and embedded in paraffin. Hearts for cryosection were embedded in tragacanth gum (Sigma), frozen in isopentane (BDH-AnalaR) cooled in liquid N_2_, cut at 9 μm sections using a cryostat (Leica CM3050), collected onto charged slides, and stored at −20 °C for histological and immunofluorescence staining. Hearts for protein and RNA extraction were snap-frozen in liquid N_2_ and stored for further processing.

### Cardiomyocyte isolation

Cardiomyocyte isolation was performed as per standard protocol^66^. Briefly, mice were anaesthetized with isoflurane (100% O_2_ at 0.5 L/min, isoflurane atomizer dial at 4%). Hearts were stopped with 15% KCl, the descending aorta was cut, and hearts flushed with 7 mL of EDTA buffer through the right ventricle. The ascending aorta was clamped using Reynolds forceps, and the entire heart removed and placed in a 60 mm dish containing fresh EDTA buffer. Hearts were digested by sequential injection of 10 mL EDTA buffer, 3 mL perfusion buffer, and 30–50 mL collagenase buffer into the left ventricle. Forceps were used to gently pull the digested heart into smaller pieces ~1 mm and gentle trituration. Enzymatic activity was inhibited by the addition of 5 ml of Stop buffer. The cell suspension was passed through a 100 µm filter, and four sequential rounds of gravity settling to enrich for myocytes, ultimately resulting in a highly pure myocyte fraction. CMs were plated on laminin-coated coverslips and cultured for 24 h before fixation with ice-cold Methanol for further processing.

### Histological and immunofluorescence microscopy

For histological studies, sections (9 µm) were stained with standard Haematoxylin and Eosin for cell morphology, Masson’s trichrome stain to detect collagen, and TUNEL assay (Abcam) to detect apoptotic nuclei. Images were obtained with a Zeiss Axio Imager Microscope. For immunofluorescence of frozen hearts, sections were warmed to room temperature, rehydrated with PBS, blocked with M.O.M block (Vector Shields) and donkey serum (Sigma-Aldrich), incubated with primary antibodies overnight at 4 °C. The slides were then washed in PBS and incubated with secondary antibodies and Hoechst dye (Sigma-Aldrich) for 60 min, washed with PBS, and mounted in Prolong-Gold Antifade reagent (Invitrogen). Primary antibodies are listed in Supplementary Table 2 and the methods for producing in 4 house monoclonals is described in Supplementary Methods: The antibodies, their dilution and source at which used were, anti-HA epitope (rat mAB, 3F10, 1:200, Roche), LMNA/C N-18 (goat, 1:50, Santa Cruz), LaminA/C (rabbit mAB, abcam 1:200), LaminB1 conjugated to Neon-tag, mSun1 (mouse mAB, clone 12.11, neat), hSun1 monoclonal (mouse mAB, clone12.10F, neat), hSun1 (mouse mAB, clone 9.1, neat), human specific Sun1 (mouse mAB, clone 25.1, neat), Sun2 (mouse mAB, clone 3.1E, neat), Nesprin1 (mouse mAB, MANNES1E 8C3, 1:200, from Glenn E. Morris), Myosin Heavy Chain (mouse mAB, MF20, 1:50, Developmental Studies Hybridoma bank), PCM-1 (rabbit, 1:200, Sigma) and sarcomere-α-actinin (mouse mAB, EA53, 1:500, Abcam); Secondary antibodies: Alexa Fluorophore 488, 568 and 647 (1:250, Invitrogen), DAPI (1:500, Invitrogen), and Hoechst 33342 (1:5000, Invitrogen). For isolated cardiomyocyte immunofluorescence, myocytes were stained on coverslips. Permeabilization was performed with 0.1% TritonX in PBS for 20 min, followed by gentle washing with PBS, blocking with 3% BSA in 0.1% TritonX in PBS for 1 h, incubated overnight at 4 °C with primary antibody prepared in blocking solution, followed by gentle washing with PBS, then incubated with the secondary antibody for 1 h at room temperature, followed by gentle washing with PBS, and mounted with Prolong-Gold Antifade (Invitrogen). Imaging was performed within 24 h with the confocal microscope Olympus FV3000. For quantification of the intensity of Lamin A/C, Cell Profiler, and ImageJ 1.53c, VevoLab 3.2.6 software was used. To determine the level of signal displacement for Nesprin1 after upon expression ImageJ software was used. LaminB1 signal was used to define the region of interest and nuclear envelope. The mean gray value of ROI was determined for Nesprin1 and hSun1. The mean background was subtracted from each value. Only binucleated cells were chosen.

### Western analysis for LMNA, SUN1, Ha-tag, and GFP

Whole hearts were homogenized in RIPA lysis buffer and the extract spun at 13200 g, 10 min, 4 °C. Total cell lysates were electrophoresed and transferred to PVDF membrane and blocked with Odyssey Blocking Buffer (Li-Cor Biosciences). The membrane was incubated with primary antibodies for 2 h at room temperature or overnight at 4˚C, washed in TBST washing solution, and incubated in Odyssey IRDye secondary antibodies (1:5000) for 1 h before visualization with the Odyssey Infrared Imaging System (Li-Cor Biosciences). The primary antibodies used: for detection of LaminA/C (Rabbit, 1:500, Cell Signaling) that is specific to an epitope in the first 50 amino acids in LMNA, mSun1 (mouse mAB, clone 12.11, neat, from B. Burke), GFP (mouse, 1:500, Roche), LaminB1 (rabbit mAB, 1:500, Abcam), anti-HA epitope (rat mAB, 3F10, 1:1000, Roche), GAPDH (rabbit, 1:500, Abcam), and control β-tubulin (mouse, Tub 2.1, 1:1000, Sigma). For detection of the AAV9-DNhSun1 transgene, a mouse mAB specific to the C-terminus of human Sun1 (hSun1, clone 9.1, neat, from B. Burke) was used in combination with protein A conjugated to HRP (1:500, Cell Signaling). GAPDH (rabbit, 1:500, Abcam) was used with anti-rabbit Immunoglobulins/HRP (DAKO, 1:2500) as a loading control. Uncropped and unprocessed scans of blots are in the Source Data file.

### Echocardiography

Cardiac function was measured by echocardiography using the Vevo2100 and Vevo3100 (VisualSonics). Mice were shaved 1 day before ultrasound examination. The animals were anaesthetized with 1.5% isoflurane mixed with oxygen. Readings of B-mode and M-mode were taken at heart rates between 450 bpm and 350 bpm. FS, EF, and GLS were calculated from the parasternal long axis using the Vevostrain feature of the VevoLab software (VisualSonics). Cardiac measurements of the left ventricular interior diameter, interventricular septum, and left ventricular posterior wall were taken from the parasternal short-axis for the diastolic and systolic state.

### Active force measurement of the cardiac papillary muscle

Mouse papillary muscle from the left ventricle was prepared according to the methods described before^67^. Briefly, the explanted mouse heart was immediately rinsed with oxygenated ice-cold Krebs–Henseleit solution with 12 U/mL heparin sodium (EDQM) and 30 mM 2,3-Butanedione monoxime BDM (Sigma) and excess blood removed. Hearts were then transferred to ice-cold Krebs–Henseleit solution in a glass petri-dish under a dissection microscope with a cooling stage. Cylindrical papillary muscle (200–300 µm in diameter and 1.5–2 mm in length) were excised from the left ventricle. T-shaped aluminum clips with a hole were crimped onto the ends of a papillary preparation and the prepared papillary chunks fixed using pins onto a glass petri-dish with a layer of PDMS sylgard 184 (Dow Corning). Papillary preparations were immersed in a 2% Triton X-100 solution at 4 °C overnight. Force measurements were performed as previously described^68^. The T-shaped aluminum clips at the ends of the papillary preparations were attached to the hooks of a force transducer (AE801, HJK Sensoren+Systeme) and servo-motor in the experimental rig was glued with shellac in ethanol (Sigma) to minimize the movement during the experiment. Papillary contraction force was measured at 20 °C. The maximum contraction force was measured inactivating solution (100 mM TES, 6.5 Mm MgCl_2_, 25 mM Ca-EGTA, 5.7 mM Na_2_ATP, 20 mM glutathione, 21.5 mM sodium creatine phosphate, pH = 7.1, Ionic strength 150 mmol/L) with 32 µmol/L free Ca^2+^. Data were collected and processed from the force transducer and DAQ data acquisition device (National Instrument) using a customized software programmed by LabVIEW 2013 (National instrument). At least 5 fibers were tested from each mouse, and at least 3 mice were tested for each experimental group.

### Derivation of human-induced pluripotent stem cells (iPSCs)

Human iPSCs were generated from a healthy male patient^69^ using the episomal reprogramming method^70^. Informed consent was obtained for this procedure with UCSF Committee on human research #10-02521, which approved the study protocol. The human iPS cell lines used in this study were generated from a healthy male patient, WTC10 and WTC11. Pluripotent stem cells were maintained on Matrigel (BD Biosciences) coated polystyrene culture plates in StemFlex medium (Thermo Fisher Scientific). Cells were supplemented with Y-27632 (10 μM) (StemCell Technologies), a Rho-associated kinase (ROCK) inhibitor after passaging to promote cell survival.

### Directed cardiomyocyte differentiation from human pluripotent stem cells

Differentiation of human iPSCs to cardiomyocytes (iPSC-CMs) was performed by modulating WNT/β-catenin signaling as described (the GiWi protocol)^71^. Human pluripotent stem cells were seeded at 5 × 10^4^ cells/cm^2^ onto 12-well plates coated with Matrigel (BD Biosciences) in StemFlex medium for 3 days. On the day of differentiation, the medium was switched to RPMI medium supplemented with B27 without insulin (RPMI/B27−) (Life Technologies) and CHIR99021 (12 μM) (Tocris) for exactly 24 hr before replacing with fresh RPMI/B27− medium. After 48 h, cells were treated with IWP2 (5 μM) (Tocris) in RPMI/B27− for 2 days before replacing with fresh RPMI/B27− medium. After 2 days, the medium was then switched and maintained in RPMI/B27 with insulin (RPMI/B27+) for 8–11 days before using a metabolic selection protocol to purify iPSC-CMs^72^. Cells were re-plated and maintained on RPMI/B27+ medium for 4 days and then replaced with lactate medium (glucose-free DMEM containing sodium pyruvate and buffered lactate (4 mM) supplemented with Glutamax and nonessential amino acids). Cells were treated with lactate medium twice, with each treatment lasting for 2 days. The purified iPSC-CMs were then maintained in RPMI/B27+ medium for 1 week before harvesting for further analyses.

### AAV9-DNSUN1 and AAV9-GFP virus

The DNSUN1 (SS–HA-Sun1L–KDEL) and GFP (SS-GFP–KDEL) vectors as described^22^. Briefly, almost the entire luminal domain of Sun1 was tagged at its NH_2_ terminus with HA (HA-Sun1L). To introduce the HA-Sun1L as a soluble form into the lumen of the ER and PNS, signal sequence and signal peptidase cleavage sites of human serum albumin was fused to the NH_2_ terminus of HA-Sun1L to yield SS–HA-Sun1L. To prevent its secretion, a KDEL tetrapeptide was fused to the COOH terminus of SS–HA-Sun1L to form the final SS–HA-Sun1L–KDEL. The HA-Sun1L region was replaced with a GFP sequence to generate the SS-GFP–KDEL. The DN-Sun1 and GFP fragments were amplified with the primers listed below (same forward primer was used for both fragments, and details are provided in Supplementary Table 1) and ligated into Penn-AAV-cTnT-PI-eGFP plasmid (kind gift from J. Jian), digested with NcoI and KpnI. All restriction enzymes were purchased from NEB. PCR reactions were performed using Q5^®^ Hot Start High-Fidelity 2X Master Mix (NEB, M0494L). Ligations were performed using isothermal assembly with NEBuilder^®^ HiFi DNA Assembly Master Mix (NEB, E2621L). Primers used for constructing the plasmids were ordered from IDT.

### Production of AAV for SUN1 dominant-negative treatments

AAV Virus was produced as per standard protocol^73^. 293T cells were expanded in 15 cm dishes or T-flasks before being seeded into a 10-chamber CellSTACK (Corning Inc., Corning, NY, USA). pAAV2/9 (gift of J. M. Wilson, UPenn Vector Core, Philadelphia, PA), pHelper (Part No. 340202, Cellbiolabs, Inc., San Diego, CA, USA), and plasmids containing the transgenes (Penn-AAV-cTnT-EGFP-RBG and pAAV-cTnT-Myc-SUN1DN) were transfected using PEI Max (Polysciences, Warrington, PA, USA). 4 days after transfection, the cell pellet and supernatant were harvested. The supernatant was clarified by filtration and applied by gravity flow to ~1 ml POROS™ CaptureSelect™ AAV9 Affinity Resin (Thermo Fisher Scientific, Waltham, MA, USA). Cells were resuspended in lysis buffer (phosphate-buffered saline, 200 mM NaCl, 0.001% Pluronic F-68), lysed by 4–5 rounds of freeze/thaw, sonicated, and treated with benzonase to shear and digest DNA. Cell debris was pelleted by centrifugation, and cell lysates were collected and filtered through 0.45 μm syringe filters. Filtered lysates were then applied to AAV9 affinity resin by gravity flow. Following washing in wash buffer (phosphate-buffered saline, 500 mM NaCl), AAV9 virions were eluted using 100 mM glycine, pH 2.5, and collected in microfuge tubes containing 1/10th volume of 1 M Tris, pH8. Following 2 rounds of buffer exchange into PBS containing 0.01% Pluronic F-68 and concentration via Amicon^®^ Ultra 100 KDA concentrators (Merck KGaA, Darmstadt, Germany), ~1 ml solution containing AAV virions was filtered through 0.22 μm 4 mm Millex syringe filter units (Merck KGaA, Darmstadt, Germany) and stored in 4 °C or −80 °C. To quantify viral genomes, dye-based quantitative real-time PCR was performed using primers 5′-acagtctcgaacttaagctgca-3′ and 5′-gtctcgacaagcccagtttcta-3′, PacI-digested and PCR-purified PENN-AAV-cTnT-EGFP-RBG to generate a standard curve, and PerfeCTa SYBR Green FastMix Low ROX qPCR master mix (Quanta BioSciences, Beverly, MA, USA).

### In vivo gene delivery

Initially, the following protocol was used for AAV transduction into the mouse hearts. Mice were genotyped 10 days postnatally. They were then subjected to one IP injection of Tmx (40 mg/kg) at 14 days postnatally, followed by a single injection at a concentration of 5 × 10^10^ VG/g AAV9-DNmSun1 or AAV9-GFP virus into the thoracic cavity at 15 days postnatally. Adult mice (3–5 months old) were injected IP with a single dose of Tmx (40 mg/kg), followed by injection of AAV9 at a concentration of 5 × 10^10^ VG/g AAV9-DNmSun1 or AAV9-GFP virus into the thoracic cavity. Young and adult mice were anesthetized with a gaseous mixture of 1.5% Isoflurane (BioMac) and 1.5 L O_2_ before injections.

In the subsequent set of AAV transductions, the route of administration was changed from intrathoracic to retro-orbital injection^37^. For evaluation of the cardiac function, only male animals were used. On postnatal day 10 mice were genotyped. To induce deletion of the *Lmna* gene in CMs, a single IP injection of tamoxifen (40 mg/kg) was administered on postnatal day 14 to all animals. On postnatal day 15, animals were injected retro-orbitally with AAV9 DNhSUN1 or AAV9 GFP (up to 2 × 10^10^ VG/g, 5 ml/g) into the retro-orbital sinus using an insulin syringe. For the dose–response experiments, 5 × 10^10^ VG/g was used for the high dose. Animals were selected randomly. TMX and AAV9 injections were performed under anesthesia using 1.5% Isoflurane mixed with oxygen. The AAV9 working solution was prepared freshly before administration. Depending on the concentration of viral genomes, the respective AAV9 stock solutions were diluted with PBS containing 0.001% Pluronic F-68.

### Statistical analysis

All statistical analyses were performed using Excel 2016 and Graphpad Prism 9.1.0. Results are shown as mean with ±SD. Data were analyzed using One-way ANOVA or unpaired *T*-test as indicated. For lifespan analysis, significance was tested with Log-rank test. To calculate the significance of cardiac data Tukey’s post hoc test was used for multiple groups.

### Reporting summary

Further information on research design is available in the Nature Research Reporting Summary linked to this article.

## Supplementary information

Supplementary Information

Reporting Summary

## Data Availability

The data supporting the conclusions of this paper are provided in the article and the Supplementary Information. Any remaining raw data will be available from the corresponding author upon reasonable request. Source Data are provided with this paper.

## Source data

Source Data
