# Supplementary material for: Disrupting the LINC complex by AAV mediated gene transduction prevents progression of Lamin induced cardiomyopathy
Source: Nat Commun. 2021 Aug 5;12:4722. doi: 10.1038/s41467-021-24849-4 (PMC8342462; doi:10.1038/s41467-021-24849-4)
Supplement: Supplementary file 1 — Supplementary Information [file 41467_2021_24849_MOESM1_ESM.pdf]

Supplementary information

Disrupting the LINC complex by AAV mediated gene transduction prevents progression of Lamin induced cardiomyopathy.

Chai *et al*

Supplementary Figure 1

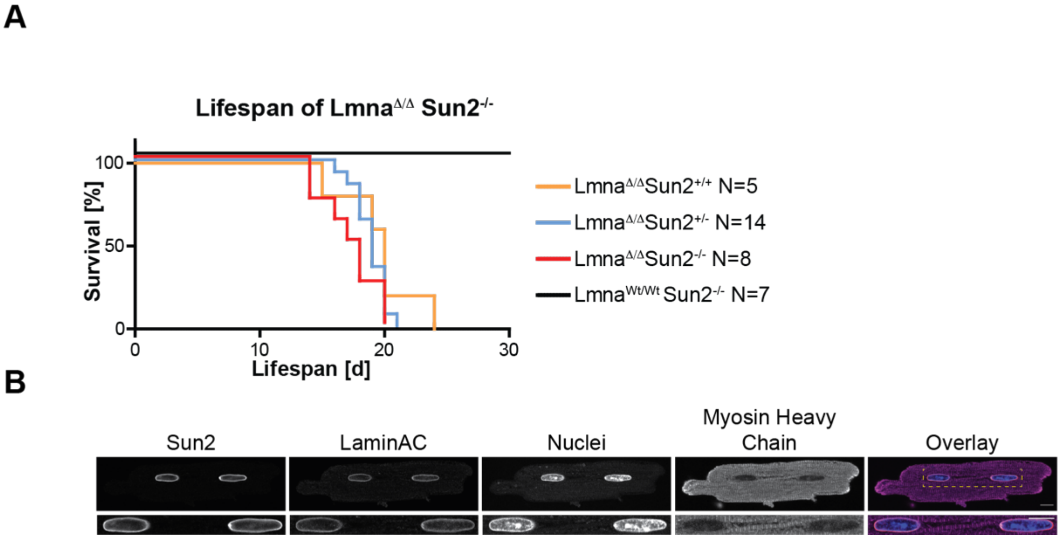

Supplementary Figure 1: Loss of Sun2 does not rescue lethality caused by *Lmna* loss. (A) Loss of Sun2 despite being expressed in CMs does not extend the lifespan of *Lmna*$^{\Delta/\Delta}$ mice. This maybe because Sun1 interacts directly with Lamin A whereas Sun2 does not or shows reduced interaction with Lamin A[36]. (B) Sun2 expression in isolated CMs from 2 month-old WT mice. Image is representative for N= 2. Myosin heavy chain is a marker for CMs. Scale bar 10μm.

Supplementary Figure 2

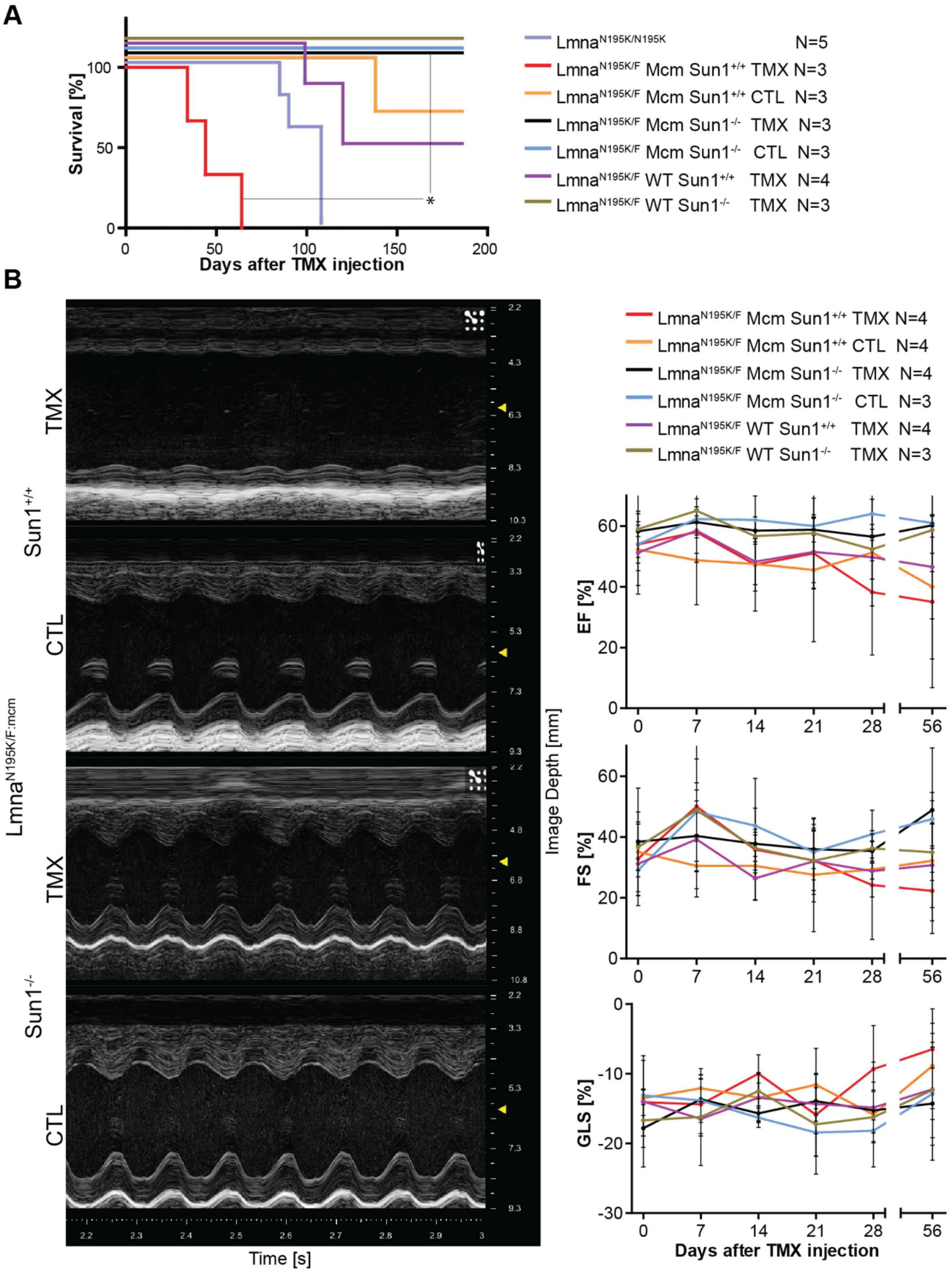

Supplementary Figure 2: Deletion of SUN1 ameliorates cardiac pathology induced by a missense mutation in the *Lmna* gene (N195K). (A) The absence of Sun1 significantly increases the lifespan of *Lmna*$^{N195K/F:mcm}$/*Sun1*$^{-/-}$ mice compared *to* *Lmna*$^{N195K/F:mcm}$/*Sun1*$^{+/+}$ mice after Cre induction. Mice with only one copy of the N195K mutation (*Lmna*$^{N195K/–:mcm}$/*Sun1*$^{+/+}$) had an average lifespan of 47 days, approximately half the lifespan of mice homozygous i.e. with two copies of the N195K allele, whereas *Lmna*$^{N195K/–:mcm}$/*Sun1*$^{-/-}$ mice live for more than 200days. (*P=0.0101; Log-rank (Mantel Cox) test) (B) Echocardiograms performed before and after *Cre* induction revealed progressive worsening of cardiac contractility in *Lmna*$^{N195K/–:mcm}$/*Sun1*$^{+/+}$ mice compared to *Lmna*$^{N195K/–:mcm}$/*Sun1*$^{-/-}$ mice over time. Echo images were recorded at 28 days after Cre induction. Data were analysed from total number of animals (N) per genotype as indicated in the graph. Data are presented as mean ±SD.

Supplementary Figure 3

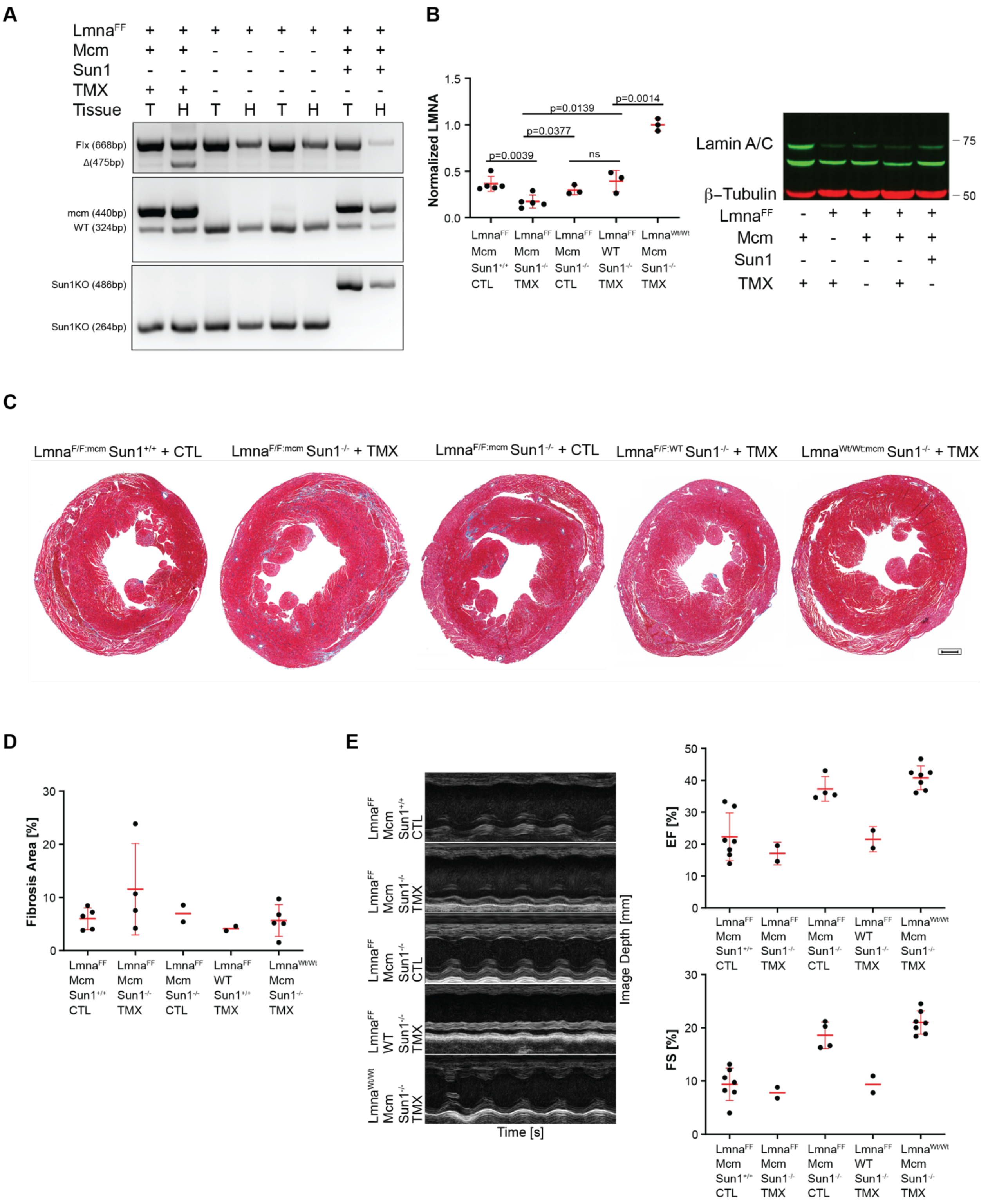

Supplementary Figure 3: Phenotypes of *Lmna*$^{F/F:mcm}$ *Sun1*$^{+/+}$ and *Lmna*$^{F/F:mcm}$ *Sun1*$^{–/–}$ hearts 12-14 months after Cre induction with Tamoxifen. (A) PCR analysis confirmed the sustained deletion of the *Lmna* gene. (**B**) Protein quantification revealed a significant reduction of LMNA levels in hearts at 14 months in *Lmna*$^{F/F:mcm}$/*Sun1*$^{–/–}$+Tmx (n=5) induced with TMX compared to *Lmna*$^{FF:mcm}$/*Sun1*$^{+/+}$ CTL (uninduced) (n=5) (. The following genotypes served as controls: *Lmna*$^{FF:mcm}$/*Sun1*$^{-/-}$ CTL (n=3), *Lmna*^{FF}/*Sun1*$^{-/-}$ +Tmx (n=3), *Lmna*$^{Wt/Wt:mcm}$/*Sun1*$^{-/-}$+Tmx (n=3). (p-values are shown in the graph, ns=not significant; unpaired two-tailed T-test), Marker in kDA. (C+D) Histology of the aged *Lmna*$^{F/F:mcm}$/*Sun1*$^{–/–}$ hearts, 12-14 months after the Tmx injection (n=4), reveal no significant (T-test) morphological changes e.g. LV enlargement or in fibrosis (blue staining) compared to the controls. The following genotypes served as controls: *Lmna*$^{FF:mcm}$/*Sun1*$^{+/+}$ CTL (n=5) ; *Lmna*$^{FF:mcm}$/*Sun1*$^{-/-}$ CTL (n=2), *Lmna*^{FF}/*Sun1*$^{-/-}$ +Tmx (n=2), *Lmna*$^{Wt/Wt:mcm}$/*Sun1*$^{-/-}$ +Tmx (n=5). Scale bar 500μm. (E) Echocardiograms from 12-14 month old mice showed reduced EF and FS in both *Lmna*$^{F/F:mcm}$ /Sun1$^{+/+}$ CTL (n=7) and *Lmna*$^{F/F:mcm}$ /*Sun1*$^{-/-}$ + Tmx (n=2) mice that was probably due to the effects of natural ageing and possible leakiness in expression of mcm. The following genotypes served as controls: *Lmna*$^{FF:mcm}$/*Sun1*$^{-/-}$ CTL (n=4), *Lmna*^{FF}/*Sun1*$^{-/-}$ +Tmx (n=2), *Lmna*$^{Wt/Wt:mcm}$/*Sun1*$^{-/-}$ +Tmx (n=7). (B+D+E) Data are shown as mean ±SD.

Supplementary Figure 4

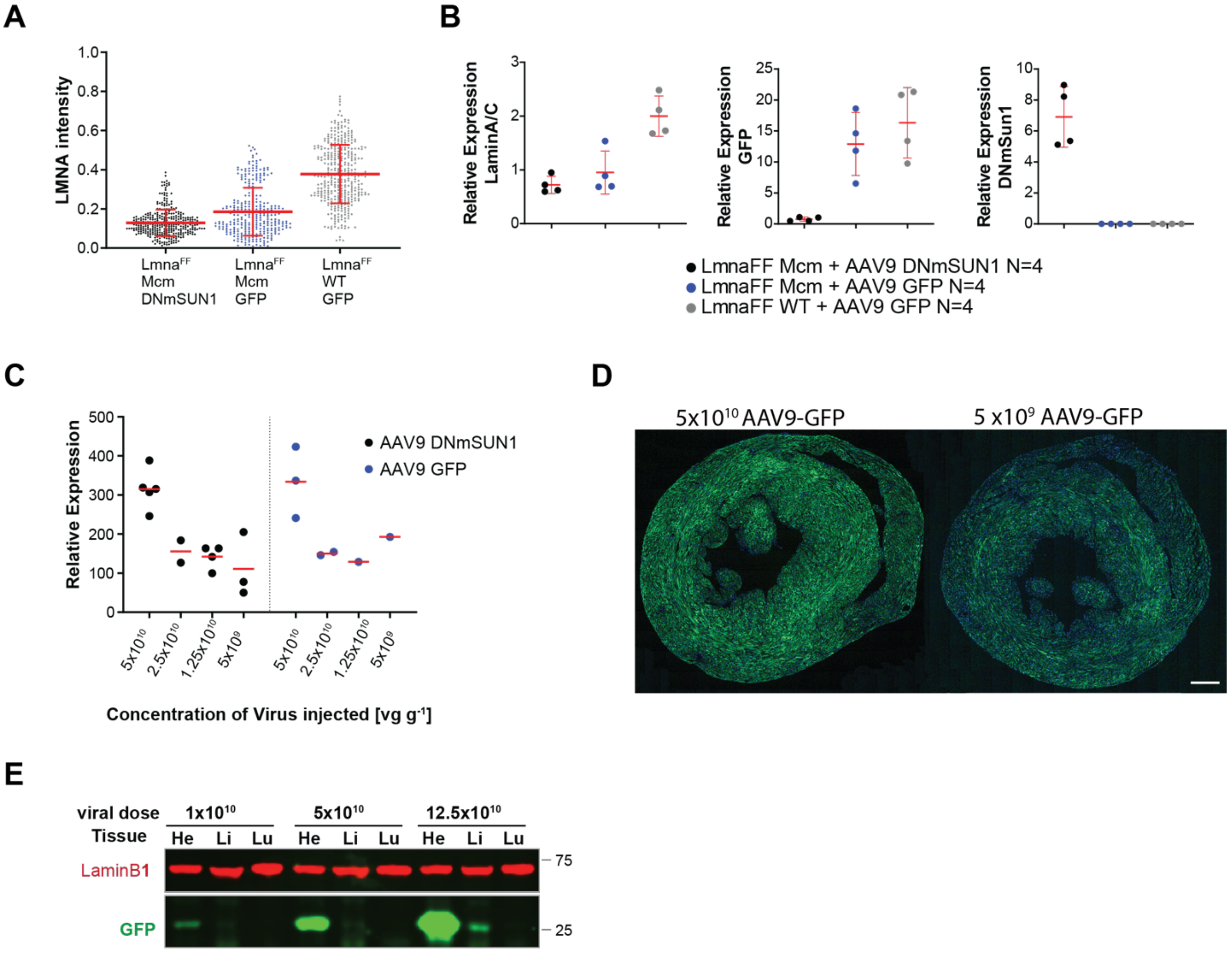

Supplementary Figure 4 AAV infection efficiency and expression levels of LaminA and Sun1. (A). Although LaminA/C levels fall following TMX injection, the presence of either AAV9-DNSUN1 (n=559) or AAV9-GFP (n=263) protein does not alter LMNA protein levels in cardiomyocytes from *Lmna*$^{FF:mcm}$ animals. Cardiomyocytes from *Lmna*^{FF} with AAV9-GFP (n=209) served as a control (Quantification of LaminA/C immunofluorescence intensity). (B) The levels of LaminA/C, DNSUN1 and GFP protein in whole hearts were quantified by Western analysis 35 days after TMX injection. Expression was normalized against β-Tubulin. (**A+B**) Data are shown as mean ±SD. (C) Expression levels of both DNSUN1 and GFP proteins are dependent on the concentration of viral particles injected.

Data are shown as mean. (AAV9 DNmSUN1: $5x10^{10}$ (n=5), $2.5x10^{10}$ (n=2), $1.25x10^{10}$ (n=4), $5x10^{9}$ (n=3); AAV9 GFP: $5x10^{10}$ (n=3), $2.5x10^{10}$ (n=2), $1.25x10^{10}$ (n=1), $5x10^{9}$ (n=1). (D) Immunofluorescence revealed CMs were extensively infected and expressed GFP with $5x10^{10}$ vg g^{-1} of AAV9-GFP compared to a 10-fold lower ($5x10^{9}$ AAV9-GFP) dosage of viral particles. vg $*g^{-1}$ = viral genome per g bodyweight. Scale bar 500μm. **(E)** The AAV9 transduced GFP is transcribed from the cTnT promoter and expression is primarily restricted to the heart (He) with expression levels varying according to dose injected. Low level expression is only detected in the liver (Li) at very high concentrations of injected AAV, and are not detected in lung (Lu). Western blot analysis is representative for 3 blots. Marker in kDA.

Supplementary Figure 5

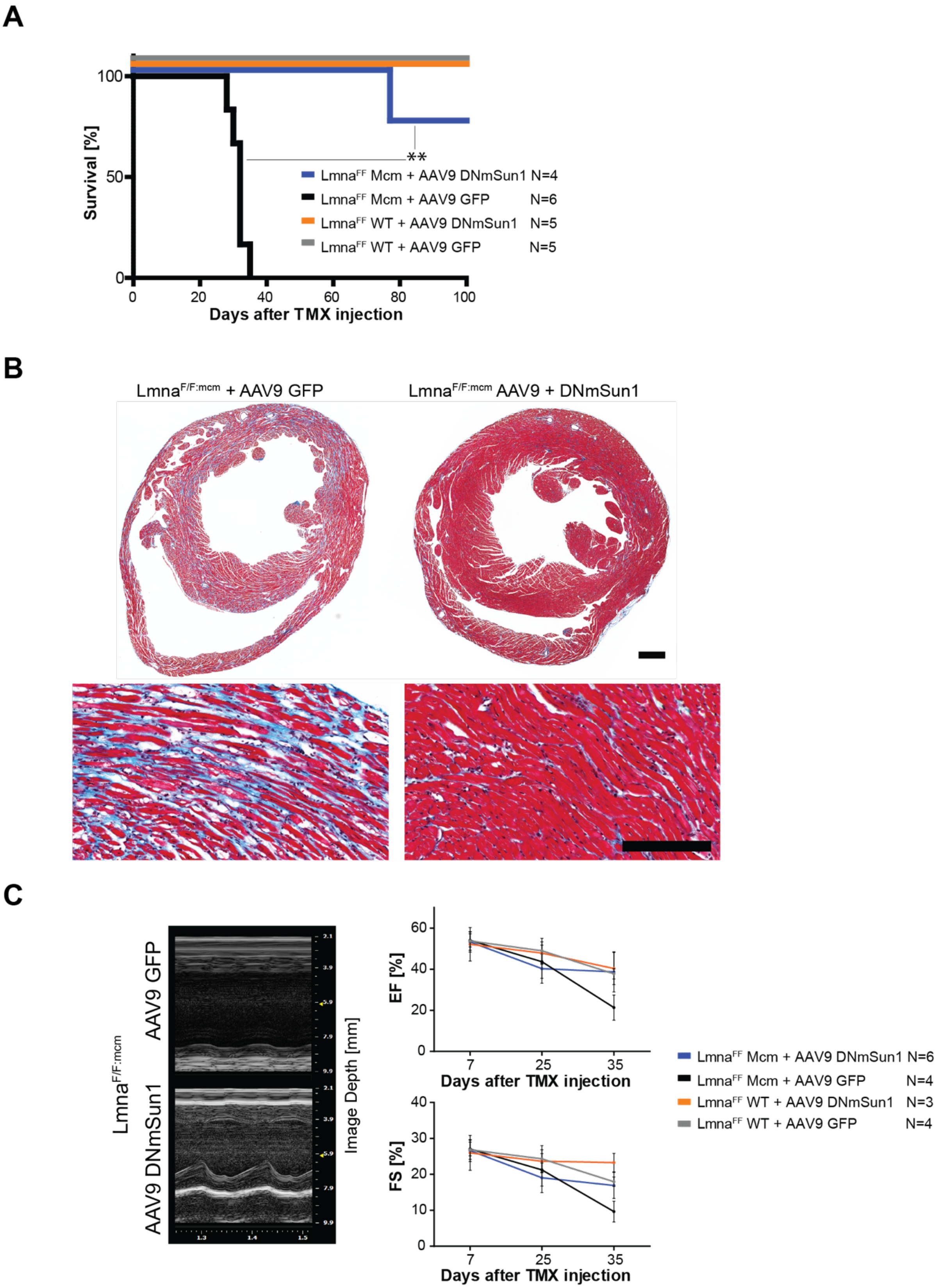

Supplementary Figure 5 *Lmna*$^{F/F:mcm}$ mice expressing AAV transduced murine DNmSUN1 have improved cardiac function and extended longevity. (A) The *Lmna*$^{F/F:mcm}$ +AAV9-GFP mice live for an average of 34.5 days after Tmx induction, whereas *Lmna*$^{F/F:mcm}$ mice injected with AA9-DNmSUN1 ($5x10^{10}$ vg g^{-1} per mouse) live significantly longer to at least 100 days post Tmx induction, after which the mice were sacrificed for analysis. (**P=0.0038, *P=0.0101; Log-rank (Mantel Cox) test). (B) At 35 days after Tmx induction, extensive fibrosis (blue) was detected in *Lmna*$^{F/F:mcm}$ +AAV9-GFP hearts compared to *Lmna*$^{F/F:mcm}$ +AAV9-DNmSUN1 hearts. Scale bar 500μm (upper panel), 100μm (lower panel). (C) Echo analysis confirmed *Lmna*$^{F/F:mcm}$ +AAV9-DNmSUN1 hearts had improved cardiac function compared to the *Lmna*$^{F/F:mcm}$ +AAV9-GFP hearts at 35 days after Tmx injection. Data were analysed from total number of animals (N) per genotype as indicated in the graph. Data are presented as mean ±SD.

Supplementary Figure 6

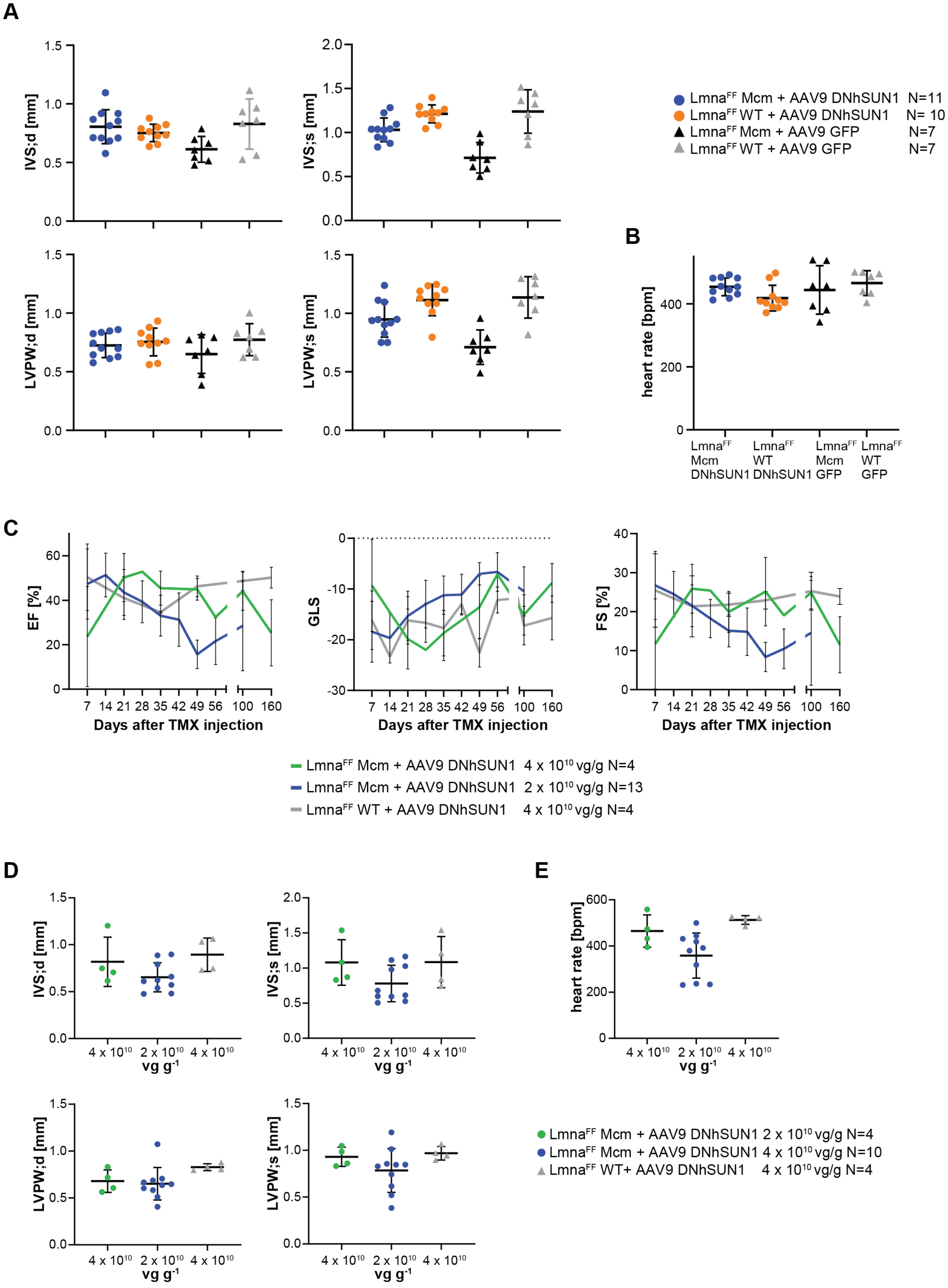

Supplementary Figure 6 Delivery of the hSUN1 dominant negative construct via AAV9 improves cardiac morphology and cardiac function (A**)** Cardiac measurements of interior ventricular septum (IVS) and left ventricular wall (LVPW) in diastole (d) and systole (s) at day 28 after Tmx injection. The wall thickness is retained in *Lmna*$^{F/F:mcm}$ + AAV9-DNhSUN1 hearts compared to the DCM model *Lmna*$^{F/F:mcm}$ + AAV9-GFP. **(**B**)** Heart rates at day28 after Tmx injection. **(**C**)** Cardiac function is improved by doubling the concentration of the DNhSUN1AAV. Echocardiogram reveals an improvement of Fractional Shortening (FS), Global Longitudinal Strain (GLS) and Ejection Fraction in *Lmna*$^{F/F:Mcm}$ animals injected with a double dose (4x10^{10}viral genomes/g bodyweight [vg/g]) of AAV9-DNhSUN1 compared to animals transduced with the standard dose (2x10^{10}vg/g). **(**D**)** Measurements of interior ventricular septum (IVS) and left ventricular wall (LVPW) in diastole (d) and systole (s) at day 56 after Tmx injection. Wall thickness is retained with the higher dose. **(E)** Heart rate at day 56 after Tmx injection. (A-E) Data were analysed from total number of animals (N) per genotype as indicated in the graphs. Data are presented as mean ±SD.

Supplementary Figure S6

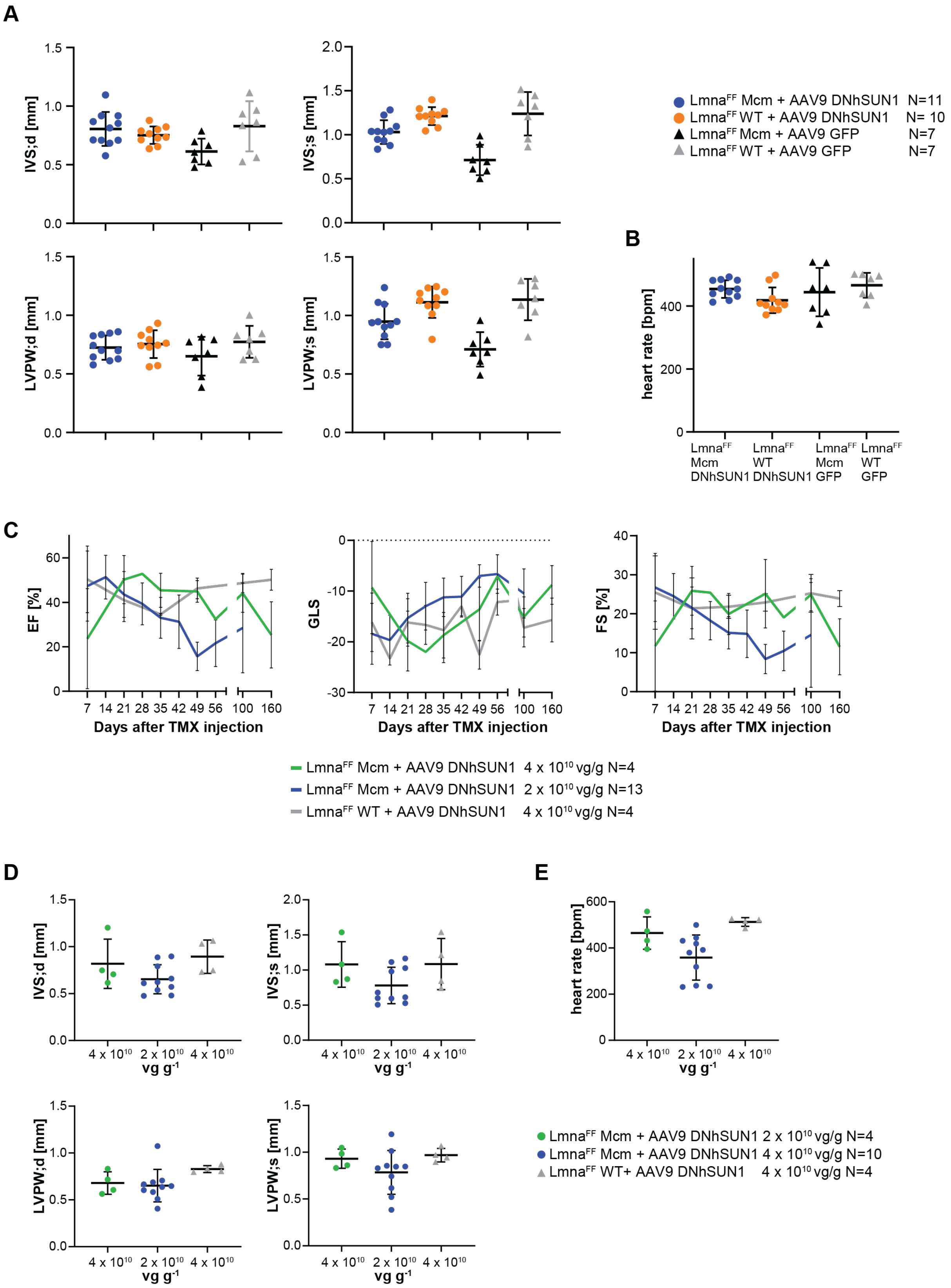

Supplementary Figure 6 Delivery of the hSUN1 dominant negative construct via AAV9 improves cardiac morphology and cardiac function (A**)** Cardiac measurements of interior ventricular septum (IVS) and left ventricular wall (LVPW) in diastole (d) and systole (s) at day 28 after Tmx injection. The wall thickness is retained in *Lmna*$^{F/F:mcm}$ + AAV9-DNhSUN1 hearts compared to the DCM model *Lmna*$^{F/F:mcm}$ + AAV9-GFP. **(**B**)** Heart rates at day28 after Tmx injection. **(**C**)** Cardiac function is improved by doubling the concentration of the DNhSUN1AAV. Echocardiogram reveals an improvement of Fractional Shortening (FS), Global Longitudinal Strain (GLS) and Ejection Fraction in *Lmna*$^{F/F:Mcm}$ animals injected with a double dose (4x10^{10}viral genomes/g bodyweight [vg/g]) of AAV9-DNhSUN1 compared to animals transduced with the standard dose (2x10^{10}vg/g). **(**D**)** Measurements of interior ventricular septum (IVS) and left ventricular wall (LVPW) in diastole (d) and systole (s) at day 56 after Tmx injection. Wall thickness is retained with the higher dose. **(E)** Heart rate at day 56 after Tmx injection. (A-E) Data were analysed from total number of animals (N) per genotype as indicated in the graphs. Data are presented as mean ±SD.

Supplementary Table 1

Primer	Sequence
**Genotyping**	
LmnaFF -F1	CCAGCTTACAGAGCACCGAGCT
LmnaFF -F2	TCCTTGCAGTCCCTCTTGCATC
LmnaFF -R1	AGGCACCATTGTCACAGGGTC
Sun1-F	GGCAAGTGGATCTCTTGTGAATTCTTGAC
Sun1-R	GTAGCACCCACCTTGGTGAGCTGGTAC
Sun1-E8	AGCCACATAACCACCTGGAG
MyHC-tF	ATGACAGACAGATCCCTCCTATCTCC
MyHC-tR	CTCATCACTCGTTGCATCATCGAC
MyHC-F	CAAATGTTGCTTGTCTGGTG
MyHC-R	GTCAGTCGAGTGCACAGTTT
mcm-3798t	AGGTGGACCTGATCATGGAG
mcm-8346t	ATACCGGAGATCATGCAAGC
mcm-7338	CTAGGCCACAGAATTGAAAGATCT
mcm-7339	GTAGGTGGAAATTCTAGCATCATCC
Zp3Cre-F	CCATGAGTGAACGAACCTGG
Zp3Cre-R	TGATGAGGTTCGCAAGAACC
Lmna N195K WT fwd	GCGAGTGGATGCTGAGAACAGG
Lmna N195K Mut Fwd	GCGTAGTGGATGCTGAGAAGCGC
Lmna N195K Anchor rev	CTCACGCAGTTCCTGAAAGG
Sun2 10400	AGAGGGCTTTGGATCCCTTA
Sun2 10401	AACAGAGTTTCCAGGCCAAA
Sun2 oIMR7415	GCCAGAGGCCACTTGTGTAG
**Cloning**	
aav Sun1 F	CGAGAATTCACGCGGGCCGCCATGAAGTGGGTAACCTTTATTTC
aav Sun1 R	CGGGTCGACTCTAGAGGTACCTTACTACAACTCATCTTTCTGGATG
aav GFP Sun R	CGGGTCGACTCTAGAGGTACTTACTACAACTCATCTTTGGATCC
**q PCR**	
viral genome 1	ACAGTCTCGAACTTAAGCTGCA
viral genome 2	GTCTCGACAAGCCCAGTTTCTA

Supplementary Methods

Monoclonal Antibody production

Monoclonal antibody production is as described in Gimpel et al., 2017. Mice were immunized intraperitoneally with approximately 50mg of fusion protein emulsified with Freund's complete adjuvant. After three weeks an approximately 50mg boost was administered by the same route in incomplete adjuvant. After an additional three weeks, approximately 50mg was administered in PBS. Three days later, the spleen was harvested, minced finely, and passed through a 100μm cell strainer. SP2/0 myeloma cells were maintained in growth medium (Advance RPMI1640, 2mM L-glutamine, 50mM β-mercaptoethanol, 10% heat inactivated fetal bovine serum, 100 U/ml penicillin; 100 mg/ml streptomycin). Spleen cells and SP2/0 myeloma cells were washed in GKN saline solution (8g/L NaCl, 0.4g/L KCl, 3.56g/L Na2HPO4.12H2O, 0.78g/L NaH2PO4.2H2O, 2g/L Glucose). Spleen cells were fused with 1-2.5 x 10^7 myeloma cells by gradual addition to the cell pellet at 37C of 1mL 50% (w/v) polyethylene glycol (Mw ~1500) over 1min, incubation for 1min, addition over 1min each of 1mL, 2mL, 8mL, then 30mL GKN saline solution). Cells were incubated for a further 5min before pelleting at 300g for 5 min. Fused cells were distributed into 20 96-well plates in hybridoma medium (Advanced RPMI 1640, 2mM L-glutamine, 50 mM β-mercaptoethanol, 15%FBS, 20%SP2-conditioned growth medium, 100U/ml penicillin; 100mg/ml streptomycin), which was changed to HAT medium (hybridoma medium with 2x HAT supplement) the following day. After 10 days, culture supernatants were screened by immunofluorescence microscopy on NRK cells grown in 96-well plates with optical plastic bottoms. Positive hybridoma cultures were expanded in 24-well plates and single cells cloned using a flow cytometer. Spent culture medium containing antibody was employed for all further experiments.

Specificity of the antibodies was confirmed by IF and western analysis on extracts from cells derived from mice with a KO of the relevant gene and by co-IF staining on cells transfected with epitope tagged variants of the relevant proteins.

Gimpel, P. et al Curr Biol Nesprin-1α-Dependent Microtubule Nucleation from the Nuclear Envelope via Akap450 Is Necessary for Nuclear Positioning in Muscle Cells 2017 Oct 9;27(19):2999-3009.e9. doi: 10.1016/j.cub.2017.08.031. Epub 2017 Sep 28.

For lamin B1 and lamin B1 scFV (neon/scarlet)

Immunogen Glutathione-S-transferase fused to C-terminal fragment of human lamin B1 (amino acids residues 388-586)

For lamin B2 Immunogen Glutathione-S-transferase fused to C-terminal fragment of mouse lamin B2 (amino acids residues 380-596)

For hSun1 25.1 Immunogen: Maltose binding protein fused to C-terminal fragment of human Sun1 (amino acids residues 360-496)

For Sun1 X12.11 and Sun1 X15. Immunogen:15 amino acids 436-581 of Uniprot Q9D666, cloned into pGEX-4T1, GST-tagged.

Figure	Target	Antibody	clone	host	sotyp	Source	Cat. No	applicatic	dilution
Fig. 2	beta Tubulin			rabbit		abcam	ab6046	Western	1:1000
Fig. 2, 6, S2, S5	beta Tubulin	Tub 2.1		mouse	IgG1	Sigma	T4026		1:2000
Fig. 2, S2	LaminA/C			rabbit		Cell Signaling	2032S	Western	1:500
Fig. 2	PCM-1			rabbit		Sigma	HPA023374	IF, frozen	1:200
Fig. 2	Sun1 (exons 6, 8, 10)		12.10F	mouse		gift of B. Burke*		IF, frozen	neat
Fig. 2	Sarcomeric alpha-actinin	EA53		mouse	IgG1	abcam	ab9465	IF, frozen	1:100
Fig. 4	mSun1 (aa436-581)		X12.11	mouse	IgG2b	gift of B. Burke*		Western, IF	neat
Fig. 4, 6	GAPDH			rabbit		abcam	ab9485	Western	1:500
Fig. 4, 6	Nesprin1	MANNES1E	8C3	mouse	IgG1	Randles KN, et al.2010 **		IF	1:200
Fig. 4, 6, S1, S5	Nucleus	Hoechst 33342				invitrogen	H3570	IF	1:5000
Fig. 4, S5	LaminA/C	N-18		goat		Santa-Cruz Biotechnology	sc-6215	IF, frozen	1:50
Fig. 4, 6	Nucleus	DAPI				invitrogen	3571	IF, frozen	1:250
Fig. 4	Nucleus	DAPI						IF, frozen	2 mg/mL
Fig 6, S5	HA	Anti-HA	3F10	rat	IgG1	Roche	11867423001	IF, Western	1:500
Fig 6, S5	GFP	Anti-GFP	7.1, 13.1	mouse	IgG1	Roche	11814460001	Western	1:500
Fig 6	hSun1 (aa360-496)		25.1	mouse	IgG2a	gift of B. Burke*		IF	neat
Fig 6	LaminB1 (aa388-586)	LaminB1-Neon		mouse	Fvsc	gift of B. Burke*		IF	neat
Fig 6	hSun1 (aa616-812)		9.1	mouse	IgG1	gift of B. Burke*		Western	neat
Fig S1	Sun2 (aa1-212)		3.1E	mouse	IgG1	gift of B. Burke*		IF	neat
Fig S1	LaminA/C			rabbit		abam	ab133256	IF	1:200
Fig S1	Myosin Heavy Chain	MF20		mouse	IgG2b	Developmental Studies Hybridoma Bank	MF20	IF	1:50
Fig S5	Lamin B1			rabbit		abcam	ab133741	Western	1:500
Fig 6	Protein A	Protein A HRP conjugated				Cell Signaling	12291	Western	1:500
Fig 6	Anti-Rabbit Immunoglobulins/HRP			goat		Dako	P0448	Western	1:2500
	Alexa 488, 568, 6	anti IgG1		goat		Invitrogen	A21121,A21124,	IF	1:500
	Alexa 488, 568, 6	IgG2a		goat		Invitrogen	A21131, A21134	IF	1:500
	Alexa 488, 568, 6	IgG2b		goat		Invitrogen	A21141, A21144	IF	1:500
	Alexa 647	anti IgG1		goat		Invitrogen	A21240	IF	1:250
	Alexa 647	IgG2a		goat		Invitrogen	A21241	IF	1:250
	Alexa 647	IgG2b		goat		Invitrogen	A21242	IF	1:250
	Alexa 488, 568,	anti-rabbit		goat		Invitrogen	A11034, A11036	IF	1:500
	IRDye 800CW	anti-rabbit, anti-mouse		donkey		Licor	926-32211,926-32210	Western	1:5000
	IRDye 680RD	anti-rabbit, anti-mouse		donkey		Licor	926-68071,926-68072	Western	1:5000

Randles KN, et al. Dev Dyn. 2010;239(3):998-1009. doi:10.1002/dvdy.22229
Gimpel P, et al. Curr Biol. 2017;27(19):2999-3009.e9. doi:10.1016/j.cub.2017.08.031
