# Supplementary material for: Disrupting the LINC complex by AAV mediated gene transduction prevents progression of Lamin induced cardiomyopathy
Source: Nat Commun. 2021 Aug 5;12:4722. doi: 10.1038/s41467-021-24849-4 (PMC8342462; doi:10.1038/s41467-021-24849-4)
Supplement: Supplementary file 2 — Reporting Summary [file 41467_2021_24849_MOESM2_ESM.pdf]

nature research

Corresponding author(s): Colin L Stewart, R S Foo

Last updated by author(s): 2021/06/26

Reporting Summary

Nature Research wishes to improve the reproducibility of the work that we publish. This form provides structure for consistency and transparency in reporting. For further information on Nature Research policies, see our Editorial Policies and the Editorial Policy Checklist.

Statistics

For all statistical analyses, confirm that the following items are present in the figure legend, table legend, main text, or Methods section.

n/a	Confirmed	
☐	☒	The exact sample size (n) for each experimental group/condition, given as a discrete number and unit of measurement
☐	☒	A statement on whether measurements were taken from distinct samples or whether the same sample was measured repeatedly
☐	☒	The statistical test(s) used AND whether they are one- or two-sided<br>*Only common tests should be described solely by name; describe more complex techniques in the Methods section.*
☐	☒	A description of all covariates tested
☒	☐	A description of any assumptions or corrections, such as tests of normality and adjustment for multiple comparisons
☐	☒	A full description of the statistical parameters including central tendency (e.g. means) or other basic estimates (e.g. regression coefficient) AND variation (e.g. standard deviation) or associated estimates of uncertainty (e.g. confidence intervals)
☐	☒	For null hypothesis testing, the test statistic (e.g. F, t, r) with confidence intervals, effect sizes, degrees of freedom and P value noted<br>*Give P values as exact values whenever suitable.*
☒	☐	For Bayesian analysis, information on the choice of priors and Markov chain Monte Carlo settings
☒	☐	For hierarchical and complex designs, identification of the appropriate level for tests and full reporting of outcomes
☒	☐	Estimates of effect sizes (e.g. Cohen's d, Pearson's r), indicating how they were calculated

Our web collection on statistics for biologists contains articles on many of the points above.

Software and code

Policy information about availability of computer code

Data collection	GraphPad Prism 9.1.0, Excel 2016.
Data analysis	ImageJ 1.53c, VevoLab 3.2.6, GraphPad Prism 9.1.0, , Excel 2016.

For manuscripts utilizing custom algorithms or software that are central to the research but not yet described in published literature, software must be made available to editors and reviewers. We strongly encourage code deposition in a community repository (e.g. GitHub). See the Nature Research guidelines for submitting code & software for further information.

Data

Policy information about availability of data

All manuscripts must include a data availability statement. This statement should provide the following information, where applicable:
- Accession codes, unique identifiers, or web links for publicly available datasets
- A list of figures that have associated raw data
- A description of any restrictions on data availability

Access to the data will be provided upon reasonable request to the corresponding author

Field-specific reporting

Life sciences study design

All studies must disclose on these points even when the disclosure is negative.

Sample size	Sample sizes were increased from 3 to at least 10 or more to meet statistical criteria relating to variation and significance (p)
Data exclusions	No data was excluded
Replication	Experiments were replicated by analyzing at least 10 samples in each group (mice) and >50 samples when analyzing cellular parameters
Randomization	Samples were not randomized as specific measurements were made on mice treated with the reagent in question or control substance
Blinding	Blinding was not relevant as we were undertaking complex measurement on heart function of mice either treated with a known reagent and compared to those treated with control reagents

Reporting for specific materials, systems and methods

We require information from authors about some types of materials, experimental systems and methods used in many studies. Here, indicate whether each material, system or method listed is relevant to your study. If you are not sure if a list item applies to your research, read the appropriate section before selecting a response.

Materials & experimental systems

n/a	Involved in the study	
	x	Antibodies
	x	Eukaryotic cell lines
x		Palaeontology and archaeology
	x	Animals and other organisms
x		Human research participants
x		Clinical data
x		Dual use research of concern

Methods

n/a	Involved in the study	
x		ChIP-seq
x		Flow cytometry
x		MRI-based neuroimaging

Antibodies

Antibodies used

Figure Target Antibody clone host Isotype Source Cat. No application dilution
Fig. 2 beta Tubulin rabbit abcam ab6046 Western 1:1000
Fig. 2, 6, S2, S5 beta Tubulin Tub 2.1 mouse IgG1 Sigma T4026 1:2000
Fig. 2, S2 LaminA/C rabbit Cell Signaling 2032S Western 1:500
Fig. 2 PCM-1 rabbit Sigma HPA023374 IF, frozen 1:200
Fig. 2 Sun1 (exons 6, 8, 10) 12.10F mouse gift of B. Burke* IF, frozen neat
Fig. 2 Sarcomeric alpha-actinin EA53 mouse IgG1 abcam ab9465 IF, frozen 1:100
Fig. 4 mSun1 (aa436-581) X12.11 mouse IgG2b gift of B. Burke* Western, IF neat
Fig. 4, 6 GAPDH rabbit abcam ab9485 Western 1:500
Fig. 4, 6 Nesprin1 9F10 9F10 mouse IgG1 Ximbio 153480 IF neat
Fig. 4, 6, S1, S5 Nucleus Hoechst 33342 invitrogen H3570 IF 1:5000
Fig. 4, S5 LaminA/C N-18 goat Santa-Cruz Biotechnology sc-6215 IF, frozen 1:50
Fig. 4, 6 Nucleus DAPI invitrogen 3571 IF, frozen 1:250
Fig. 4 Nucleus DAPI IF, frozen 2 mg/mL
Fig 6, S5 HA Anti-HA 3F10 rat IgG1 Roche 11867423001 IF, Western 1:500
Fig 6, S5 GFP Anti-GFP 7.1, 13.1 mouse IgG1 Roche 11814460001 Western 1:500
Figs 6, 7 hSun1 (aa360-496) 25.1 mouse IgG2a gift of B. Burke* IF neat
Fig 6 LaminB1 (aa388-586) LaminB1-Neon mouse Fvsc gift of B. Burke* IF neat
Fig 6 hSun1 (aa616-812) 9.1 mouse IgG1 gift of B. Burke* Western neat
Fig S1 Sun2 (aa1-212) 3.1E mouse IgG1 gift of B. Burke* IF neat
Fig S1 LaminA/C rabbit abam ab133256 IF 1:200
Fig S1 Myosin Heavy Chain MF20 mouse IgG2b Developmental Studies Hybridoma Bank MF20 IF 1:50
Fig S5 Lamin B1 rabbit abcam ab133741 Western 1:500

Fig 6 Protein A Protein A HRP conjugated Cell Signaling 12291 Western 1:500
Fig 6 Anti-Rabbit Immunoglobulins/HRP goat Dako P0448 Western 1:2500
Alexa 488, 568, 647, anti IgG1 goat Invitrogen A21121,A21124, IF 1:500
Alexa 488, 568, 647, IgG2a goat Invitrogen A21131, A21134 IF 1:500
Alexa 488, 568, 647 IgG2b goat Invitrogen A21141, A21144 IF 1:500
Alexa 647 anti IgG1 goat Invitrogen A21240 IF 1:250
Alexa 647 IgG2a goat Invitrogen A21241 IF 1:250

	Alexa 647 IgG2b goat Invitrogen A21242 IF 1:250<br>Alexa 488, 568, anti-rabbit goat Invitrogen A11034, A11036 IF 1:500<br><br>IRDye 800CW anti-rabbit, anti-mouse donkey Licor 926-32211,926-32210 Western 1:5000<br>IRDye 680RD anti-rabbit, anti-mouse donkey Licor 926-68071,926-68072 Western 1:5000<br><br>Gimpel P, et al. Curr Biol. 2017;27(19):2999-3009.e9. doi:10.1016/j.cub.2017.08.031
Validation	Validation for all the commercially supplied antibodies can be found at the relevant catalogue website.<br>Production of in house monoclonals, immunogens and verification is described in the Supplementary methods and below:<br>Monoclonal Antibody production<br>Monoclonal antibody production is as described in Gimpel et al., 2017. Mice were immunized intraperitoneally with approximately 50mg of fusion protein emulsified with Freund's complete adjuvant. After three weeks an approximately 50mg boost was administered by the same route in incomplete adjuvant. After an additional three weeks, approximately 50mg was administered in PBS. Three days later, the spleen was harvested, minced finely, and passed through a 100µm cell strainer. SP2/0 myeloma cells were maintained in growth medium (Advance RPMI1640, 2mM L-glutamine, 50mM b- mercaptoethanol, 10% heat inactivated fetal bovine serum, 100 U/ml penicillin; 100 mg/ml streptomycin). Spleen cells and SP2/0 myeloma cells were washed in GKN saline solution (8g/L NaCl, 0.4g/L KCl, 3.56g/L Na2HPO4.12H2O, 0.78g/L NaH2PO4.2H2O, 2g/L Glucose). Spleen cells were fused with 1-2.5 x 107 myeloma cells by gradual addition to the cell pellet at 37C of 1mL 50% (w/v) polyethylene glycol (Mw ~1500) over 1min, incubation for 1min, addition over 1min each of 1mL, 2mL, 8mL, then 30mL GKN saline solution). Cells were incubated for a further 5min before pelleting at 300g for 5 min. Fused cells were distributed into 20 96-well plates in hybridoma medium (Advanced RPMI 1640, 2mM L-glutamine, 50 mM b-mercaptoethanol, 15%FBS, 20%SP2-conditioned growth medium, 100U/ml penicillin; 100mg/ml streptomycin), which was changed to HAT medium (hybridoma medium with 2x HAT supplement) the following day. After 10 days, culture supernatants were screened by immunofluorescence microscopy on NRK cells grown in 96-well plates with optical plastic bottoms. Positive hybridoma cultures were expanded in 24-well plates and single cells cloned using a flow cytometer. Spent culture medium containing antibody was employed for all further experiments.<br>Specificity of the antibodies was confirmed by IF and western analysis on extracts from cells derived from mice with a KO of the relevant gene and by co-IF staining on cells transfected with epitope tagged variants of the relevant proteins.<br>Gimpel, P. et al Curr Biol Nesprin-1α-Dependent Microtubule Nucleation from the Nuclear Envelope via Akap450 Is Necessary for Nuclear Positioning in Muscle Cells 2017 Oct 9;27(19):2999-3009.e9. doi: 10.1016/j.cub.2017.08.031. Epub 2017 Sep 28.<br>Immunogens for making antibodies<br>For lamin B1 and lamin B1 scFV (neon/scarlet)<br>Immunogen Glutathione-S-transferase fused to C-terminal fragment of human lamin B1 (amino acids residues 388-586)<br>For lamin B2 Immunogen Glutathione-S-transferase fused to C-terminal fragment of mouse lamin B2 (amino acids residues 380-596)<br>For hSun1 25.1 Immunogen: Maltose binding protein fused to C-terminal fragment of human Sun1 (amino acids residues 360-496)<br>For Sun1 X12.11 and Sun1 X15. Immunogen:15 amino acids 436-581 of Uniprot Q9D666, cloned into pGEX-4T1, GST-tagged.

Eukaryotic cell lines

Policy information about cell lines

Cell line source(s)	HEK 293T Cell Line, HCL4517Open Biosystems, iDNA Biotechnology Pte Ltd
Authentication	The cell line was not authenticated
Mycoplasma contamination	Tested negative for mycoplasma on a monthly basis
Commonly misidentified lines (See ICLAC register)	No commonly misidentified lines were used

Animals and other organisms

Policy information about studies involving animals; ARRIVE guidelines recommended for reporting animal research

Laboratory animals	Mus musculus C57BL6 and 129J strains, Ages 0.5mths-36mths<br>Mouse lines. Mouse (C57Bl6/J and 129Sv/J) strains were maintained at the A*STAR Biological Resource Centre facility and the NUS Animal Facility on a 12 hour light/dark cycle in ventilated animal barrier facilities with the temperature set to 21 ± 1°C, humidity at 55-70% and with food and water provided ad libitum. Ethical oversight and approval were granted by the Institutional Animal Care and Use Committees, for both the ASTAR Biological Resource Centre (BRC) and the NUS AICUC and the animal facility/committee (Comparative Medicine protocol R16-213) and is governed by the association of AAALAC (USA) providing guidelines to both AALAS (USA) and AVS (Singapore) to which NUS adheres.
Wild animals	Not used
Field-collected samples	Not collected
Ethics oversight	IACUC approval from ASTAR Biological Resource Centre and NUS IACUC (see methods section) . Ethical oversight and approval were granted by the Institutional Animal Care and Use Committees, for both the ASTAR Biological Resource Centre (BRC) and the NUS AICUC and the animal facility/committee (Comparative Medicine protocol R16-213) and is governed by the association of AAALAC (USA) providing guidelines to both AALAS (USA) and AVS (Singapore) to which NUS adheres.

Note that full information on the approval of the study protocol must also be provided in the manuscript.
